# Screening and identification of protein 29 of *Echinococcus granulosus* interacting molecules

**DOI:** 10.3389/fcimb.2025.1560436

**Published:** 2025-05-02

**Authors:** Zailing Shang, Fei Qiao, Yaning Li, Xuelin Ma, Mingxia Wang, Wenji Yang, Tianyu He, Haixia Ma, Yana Wang

**Affiliations:** ^1^ Basic Medical Institute of Ningxia Medical University, Yinchuan, China; ^2^ School of Clinical Medicine of Ningxia Medical University, Yinchuan, China; ^3^ Key Laboratory of Common Infectious Diseases of Ningxia Autonomous Region, Ningxia Medical University, Yinchuan, China

**Keywords:** P29 of *Echinococcus granulosus*, immunoprecipitation, mass spectrometry, protein-protein interactions, cytoskeleton

## Abstract

**Introduction:**

Eg.P29 is a specific protein of *E.granulosus* metacestod, and has been able to induce high levels of protection in mice and sheep against challenge with protoscolex of *E.granulosus*. Therefore, Eg.P29 plays a crucial role in the growth and development of the *E. granulosus* metacestode. However, the function of Eg.P29 remains unknown. During the process life, protein is commonly with other proteins to form a complex network of interactions to play the function. In this study, we identified the interacting molecules of Eg.P29 for foundation to exploring the function of Eg.P29 in *E. granulosus* metacestode.

**Methods:**

Some basic information about Eg.P29 through bioinformatics software. A total of 50 molecules interacting with Eg.P29 were screened by three immunoprecipitation-combined liquid chromatography-tandem mass spectrometry intersections. The *Eg.P29*/pEGFP-C1 vector was transfected into cells to observe the morphological changes. Seven molecules as actin and actin-related proteins were screened using Gene Ontology and Kyoto Encyclopedia of Genes and Genomes analyses, and the effects of Eg.P29 on them were verified at the mRNA and protein levels. Interacting proteins of Eg.P29 were identified by yeast two-hybrid assay and immunoprecipitation. The localization and distribution of Eg.P29 and Eg.actin on protoscolex were observed by immunohistochemistry.

**Results:**

Eg.P29 is a soluble cytoplasmic protein containing a BAR structural domain that may play a role in actin polarity and endocytosis. Cells in the Eg.P29 group showed marked morphological changes in the form of rounded, or oval shapes, which were attributed to changes in actin distribution. Seven molecules interacting with Eg.P29 were mainly actin and actin-related proteins (ACTG1, ACTN4, VCL, ARPC1A, LIMA1, FLNB and MYH10), and their mRNA and protein levels were significantly affected by Eg.P29. Yeast two-hybridization experiments showed that VCL did not interact with Eg.P29, whereas the other molecules interacted with Eg.P29. The interaction of LIMA1 and actin with Eg.P29 was verified by co-immunoprecipitation. Additionally, Eg.P29 and Eg.actin had the same histological location on rostellum, and suckers of the protoscolex. Additionally, the seven molecules were all discovered their homologous proteins in *E.granulosus* and formed a network of interacting proteins.

**Discussion:**

In this study, we confirmed LIMA1 and actin were Eg.P29-interacting molecules, they were forming a compound regulation system to affect cytoskeleton formation. And we also deduced that Eg.P29 interacts with Eg.actin in *E. granulosus*, which collaborate to affect the activity and development of protoscolex. These aspects warrant further in-depth study.

## Graphical Abstract

By Figdraw.

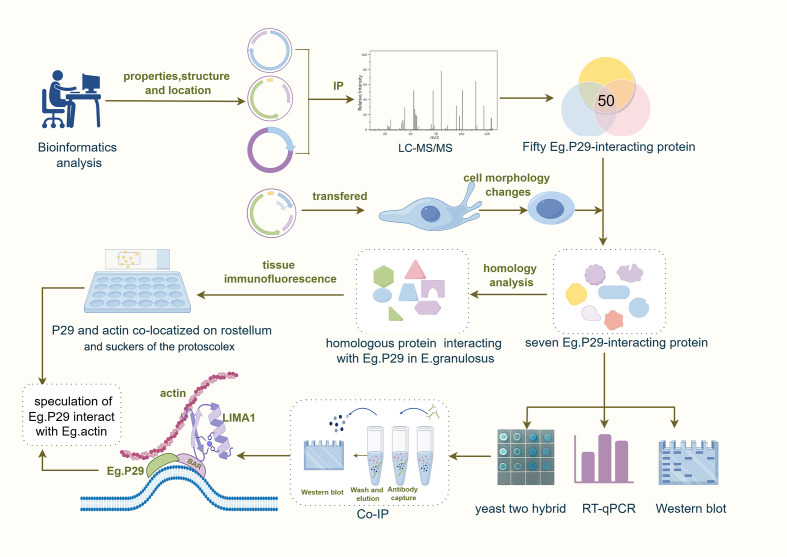

## 1 Introduction

Echinococcosis is a chronic zoonotic parasitic disease that is pervasive worldwide (McManus et al., 2012; Wen et al., 2019). The larvae of *Echinococcus granulosus* (*Eg*) and *Echinococcus multilocularis* (*Em*) both cause human echinococcosis, known as cystic echinococcosis (CE) and alveolar echinococcosis (AE). Globally, approximately two to three million patients are afflicted with CE (Craig et al., 2007). In endemic regions, the incidence of human CE can exceed 50/100,000 per year, causing a global loss of at least 1 million disability-adjusted life years (DALYs) and a financial burden of at least 76 million dollars (Budke et al., 2006). Notably, 40 % of the total number of DALYs of CE are in China (Agudelo Higuita et al., 2015), significantly affecting the population’s health and socioeconomic development. Every year, approximately $100 million is spent on controlling and treating echinococcosis, with economic losses in livestock globally ranging from $100 to $200 million (Qian et al., 2017).

The prevention and control of echinococcosis through vaccination has been unanimously recognized as an effective strategy (Zhang et al., 2003). With the application of novel immunology, cell biology, molecular biology, and other technologies, significant progress has been made in the research on vaccines against echinococcosis. Dozens of candidate molecules for echinococcosis vaccines have been cloned domestically and internationally (Zhang and McManus, 2006). Among them, Eg.P29 is an antigenic molecule identified by Gonzalez (González et al., 2000) in 1999 using a monoclonal antibody against protoscolex. It contains a 717-bp open reading frame, encodes a protein of 238 amino acid residues, and is named P29 because of its molecular weight size of 29 kDa (Shi et al., 2009). It is localized within the tegument, rostellum, and suckers of the protoscolex and germinal layers of cyst walls and is undetectable in hydatid cyst fluid and adult worm extracts (González et al., 2000; Ahn et al., 2016). Thus, it is a specific protein for the growth and development of metacestodes and is considered a potential diagnostic antigen for echinococcosis. Because it interacts with host mucosal epithelium, it may be involved in parasite escape from host immune attack, so it has received attention as a valuable vaccine molecule against *E. granulosus* (Tao et al., 2022). In our previous studies, the Eg.P29 antigen was used to immunize sheep and mice and induced greater protection in hosts against infection with *E. granulosus* (Shi et al., 2009; Hao et al., 2016; Wang et al., 2016). Another study revealed that Em.P29 exhibited promising therapeutic potential in treating mice infected with *Echinococcus multilocularis*, effectively diminishing the parasite burden in mice (Boubaker et al., 2015; Ghalia et al., 2015). These studies demonstrate that P29 molecules can exert both preventive and therapeutic effects on echinococcosis, indicating its potential utility in vaccines against hydatid disease.

Although P29 is important in *E. granulosus*, its functions are still unknown. During the process of life, the metabolism of a single protein rarely plays a significant role, and rather forms complexes with numerous other proteins to constitute a complex network of interactions and mutual regulation, known as the protein-protein interaction network (PPI) (Hu et al., 2016). Therefore, it is critical to identify the molecules that interact with Eg.P29 and investigate their relationships, which is expected to help elucidate the functions and roles of Eg.P29 in the growth and development of *E. granulsous* metacestodes.

In this study, the biological and chemical properties of Eg.P29 were comprehensively characterized using various bioinformatics software packages and websites. Four eukaryotic expression plasmids of Eg.P29 were constructed and individually transfected into cells to obtain a pool of molecules that interact with Eg.P29 via immunoprecipitation coupled with liquid chromatography-tandem mass spectrometry (LC-MS/MS) (Kaboord and Perr, 2008; Barbara and Maria, 2008). Subsequently, seven potential actin-related molecule interactors with Eg.P29 were screened, validated, and confirmed using yeast two-hybrid (Y2H) and co-immunoprecipitation (Co-IP) experiments. Finally, two protein interactors with Eg.P29 were discovered. This study provides a theoretical basis for exploring the role of Eg.P29 in the life functions of *E. granulosus* and as a potential mechanism for inducing host protection as a vaccine.

## 2 Materials and methods

### 2.1 Cells and strains

HEK293T cells and Henrietta Lacks (HeLa) cells were purchased from the Kunming Cell Bank of the Chinese Academy of Sciences. The *E.coli* BL21 strain and XL1-Blue were purchased from Beijing Hua Yueyang Biotechnology. *P29*/pET-28a was constructed by our research group and conserved in the Key Laboratory of Common Infectious Diseases of the Ningxia Autonomous Region. Myc-BioID2-MCS vector, pCMV-4flag vector, 3HA-pCDNA3.0. vector, and pEGFP-C1 vector were donated by Professor Luo from Purdue University.

### 2.2 Prediction of the basic properties, structure, and function of Eg.P29

We used a variety of online websites to predict the structure, function, and characteristics of Eg.P29. Refer to the Materials and Methods section 1.1 in **Supplementary Materials** for details.

### 2.3 Homology analysis

We conducted a search for Eg.P29 homologous sequences in the National Center for Biotechnology Information (NCBI, https://www.ncbi.nlm.nih.gov/), the top 20 species with the highest homology were screened for multi-sequence comparisons, and a phylogenetic tree was constructed using MEGA11. Refer to **Supplementary Materials** and Methods section 1.2 for details.

### 2.4 Construction of eukaryotic expression vectors

A restriction enzyme digest reaction with BamHI and SalI (Rebiosci Biotech Co., Ltd, Shanghai, China) was applied to recover *P29* gene fragments from *P29*/pET-28a plasmids. The purified target fragments were ligated into the plasmid pEGFP-C1, pCMV-4flag, Myc-BioID2-MCS, or 3HA-pCDNA3.0 using T4 DNA ligase (Rebiosci Biotech Co., Ltd, Shanghai, China.) at 16 °C overnight, and then transformed into competent *E.coli* BL21(DE3). Recombinant plasmids were extracted from bacterial colonies and identified through amplification of the *P29* gene and sent for sequencing at the Beijing Genomics Institute. All the plasmids used in this study are listed in **Supplementary Table S2**.

### 2.5 Plasmid transfection and cell culture

HEK293T and HeLa cells were cultured in Dulbecco’s modified Eagle medium (DMEM; Solarbio Science & Technology Co., Ltd., Beijing, China) containing 10 % serum (Invigentech, California, USA) and 1 % penicillin-streptomycin (Solarbio Science & Technology Co., Ltd., Beijing, China). Cells were placed in an incubator at 37 °C and 5 % CO_2_ (Thermo Fisher Scientific Co., Ltd, Massachusetts, USA, Mod:311). For transient expression of exogenous proteins in HEK293T or HeLa cells, 5 μL of Lipofectamine 3000, 5 μL of P3000 (Thermo Fisher Scientific Co., Ltd, Massachusetts, USA) and 2.5 μg of plasmids were added to cells per 6-well plate at a cell confluency of 80 %. The cell transfection rate was observed after 24 h of incubation. The cells were divided into three groups: control, empty vector, and Eg.P29 vector. Cells transfected with the pEGFP-C1 vector containing GFP showed green fluorescence under a fluorescence microscope.

### 2.6 Immunoprecipitation and co-immunoprecipitation

For cells transfected with the *P29*/Myc-BioID2-MCS and the corresponding empty vector Myc-BioID2-MCS, the old medium was discarded after 6 h and 50 μM of biotin-containing medium (#14400, Sigma-Aldrich Co., Ltd, Missouri, USA) was added for 24 h of incubation. Cells were collected and lysed using sonication with 1 mL of lysis buffer [50 mM of Tris (#T8060, Solarbio Science & Technology Co., Ltd., Beijing, China), pH 7.4, 500 mM of NaCl (#S8210, Solarbio Science & Technology Co., Ltd., Beijing, China), 0.4% of SDS (#S8010, Solarbio Science & Technology Co., Ltd., Beijing, China), 5 mM of EDTA (#E8030, Solarbio Science & Technology Co., Ltd., Beijing, China), 1mM of DTT (#D8220, Solarbio Science & Technology Co., Ltd., Beijing, China), and 1× complete protease inhibitor (#4693116001, Sigma-Aldrich Co., Ltd, Missouri, USA)] on ice until the mixtures became clear. Then, 30 μL of biotin magnetic beads were added to the solution (#20357, Thermo Fisher Scientific Co., Ltd, Massachusetts, USA) and incubated overnight at 4 °C for spinning. The following day, the beads were washed with buffer 1 (2 % SDS in ddH_2_O), buffer 2 [containing 0.1 % deoxycholate (#30970, Sigma-Aldrich Co., Ltd., Missouri, USA), 1 % Triton X-100 (#T8200, Solarbio Science & Technology Co., Ltd., Beijing, China), 500 mM of NaCl, 1mM of EDTA, and 50 mM of HEPES pH 7.5 (#H8090, Solarbio Science & Technology Co., Ltd., Beijing, China)], buffer 3 [250 mM of LiCl (#L9650, Sigma-Aldrich Co., Ltd, Missouri, USA), 0.5 % of NP-40 (#P0013F, Beyotime Biotechnology, Shanghai, China), 0.5 % of deoxycholate, 1 mM of EDTA, and 10 mM of Tris pH 8.1], and buffer 4 (50 mM Tris pH 7.4 and 50 mM NaCl) sequentially and 5×loading buffer was added after centrifugation. The protein mixtures were observed using 10 % sodium dodecyl sulfate-polyacrylamide gel electrophoresis (SDS-PAGE).

Cells were transfected with *P29*/pCMV-4flag and *P29*/3HA-pCDNA3.0 and the corresponding empty vectors pCMV-4flag and 3HA-pCDNA3.0, respectively. After 24 h, the cell culture media was discarded, and 1 mL of ice-cold lysis buffer was added [50 mM Tris-HCl pH 7.5 (#T1140, Solarbio Science & Technology Co., Ltd., Beijing, China), 150 mM NaCl, 1% Triton X-100] for incubation at 4°C for 2 min. The cell lysis solution was centrifuged for 10 min at 12,000 g, and the supernatant was transferred to another tube. This step was repeated three times. Then, 40 μL of Flag/HA beads (#A36801/26181, Thermo Fisher Scientific Co., Ltd, Massachusetts, USA) were added to the solution and rotated at 4°C overnight. The beads were collected at 1,000 g for 1 min and washed four times with cell lysis buffer at 4 °C. Afterward, 100 μL of peptide solution of Pierce™ 3x DYKDDDDK Flag/HA Synthetic Peptide (#A36805/26184, Thermo Fisher Scientific Co., Ltd, Massachusetts, USA) was added to the protein mixture for 2 h, and then was centrifuged at 1,000 g for 2 min. The supernatant was collected, 5× loading buffer was added, and the mixture was boiled for 5 min. The protein mixture was observed using 10 % SDS-PAGE. The whole cell lysate (Input) and immunoprecipitation (IP) were subjected to Western blotting to confirm the interaction of the molecules in the Co-IP.

### 2.7 Screening of protein molecules that interact with Eg.P29

#### 2.7.1 LC-MS/MS screening of protein molecules that interact with Eg.P29

The protein mixture samples (empty vector and Eg.P29 vector groups) obtained by IP were sent to the Public Experiment Platform of the First Hospital of Jilin University for LC-MS/MS detection. Protein bands were digested in gel with trypsin as previously described (Shevchenko et al., 2006). Digested peptides were analyzed by LC-ESI-MS/MS using the nanoflow reversed-phase liquid chromatography system (EASY-nLC 1200, Thermo Fisher Scientific Co., Ltd, Massachusetts, USA) coupled to the Orbitrap Exploris 240 Mass Spectrometer (Thermo Fisher Scientific Co., Ltd, Massachusetts, USA). The reverse phase peptide separation was accomplished using a trap column (75 μm ID × 2 cm) packed with 3 μm 100 Å PepMap C18 medium (#164946, Thermo Fisher Scientific Co., Ltd, Massachusetts, USA), and then separated on a reverse phase column (25 cm long × 75 µm ID) packed with 2 µm 100 Å PepMap C18 medium (#164941, Thermo Fisher Scientific Co., Ltd, Massachusetts, USA). The mass spectrometer was operated in positive ion and standard data-dependent acquisition mode with the Advanced Peak Detection function activated for the top 20n. The fragmentation of precursor ions was accomplished by HCD normalized collision energy of 30 %. The resolution of the Orbitrap mass analyzer was set to 60,000 and 15,000 for MS1 and MS2, respectively. Dynamic exclusion was set with an exclusion duration of 25 s. For protein identification, the raw data were processed with the software Proteome Discover (version 2.5). Proteome Discover was set to search with the following parameters: peptide tolerance at 10 ppm, MS/MS tolerance at 0.02 Da, carbamidomethyl (C) as a fixed modification, oxidation (M) as a variable modification, and a maximum of two missed cleavages. The false-discovery rates (FDRs) were controlled at <1 %. The results were analyzed using Num Unique and %Cov two-fold differences as screening conditions, and highly differentially expressed protein molecules in *P29*/pCMV-4flag, *P29*/Myc-BioID2-MCS, and *P29*/3HA-pCDNA3.0 group were screened by comparative analysis with the corresponding empty vector group. Differential protein molecules interacting with Eg.P29 were obtained from the intersection of the three results. The identified differential protein molecules interacting with Eg.P29 were analyzed by GO and KEGG pathway enrichment using the R4.2.2 software “clusterProfiler” and “ggplot2” packages respectively, and the differential protein molecules were functionally categorized in terms of molecular function, cellular components, biological processes, and pathway enrichment.

#### 2.7.2 Screening of actin-related protein molecules interacting with Eg.P29

Based on the cell forms that were changed in the Eg.P29 vector group, actin-related molecules were screened further. The GeneCards (http://www.genecards.org/) database was used to determine the nature and function of these molecules, and the STRING database was used to analyze the interaction molecules and remove proteins that were not in the network.

### 2.8 Western blot

Cells were transfected with different vectors for 24 h as described above. The total protein was extracted with whole cell lysate (Solarbio Science & Technology Co., Ltd, Beijing, China), separated with 10 % SDS-PAGE, and transferred to a 0.2 μM PVDF membrane (Sigma-Aldrich Co., Ltd, Shanghai, China.). The membrane was blocked with protein-free rapid blocking buffer (Shanghai Yase Biotechnology Co., Ltd., Shanghai, China) for 15 minutes at room temperature, and the different primary antibodies [β-actin monoclonal antibody, GAPDH monoclonal, Tubulin monoclonal, Vimentin monoclonal antibody, ARPC1A polyclonal antibody, EPLIN (LIMA1) monoclonal antibody, MYH10 monoclonal antibody, Filamin B (FLNB) polyclonal antibody (Proteintech Group, Inc, Wuhan, China), or α-actinin-4 antibody or vinculin antibody (Santa Cruz, Texas, USA)], was added and incubated overnight at 4 °C. The membrane was washed three times with TBS-T and incubated with sheep anti-mouse IgG-HRP for 2 h at room temperature. Protein expression was detected using an Enhanced ChemiLuminescence detection kit (KeyGen Biotech Co. Ltd., Jiangsu, China) in the dark and recorded with the ChemiDoc™Touch Imaging System (Bio-Rad Laboratories, Inc, California, USA). ImageJ software was used to quantify protein expression levels from Western blot analysis relatively.

### 2.9 Area of cells

Cells transfected with *P29*/pEGFP-C1 fluorescent plasmids or pEGFP-C1 empty plasmids were observed under a fluorescence microscope (Zeiss LSM900 Carl Zeiss AG, Oberkochen, Germany). Images were analyzed with ImageJ software. We randomly selected 50 cells from the groups. The area of each cell was measured by manually sketching the outline of the cell, and the average cell area of different groups was compared using GraphPad Prism 8 software.

### 2.10 Cell and tissue immunofluorescence

1×10^5^/mL of HeLa cells transfected with *Eg.P29*/pEGFP-C1 or pEGFP-C1 vector for 24 h were seeded onto coverslips, and fixed with 4 % paraformaldehyde (biosharp, Hefei, China) for 15 minutes, followed by permeabilization with 0.2 % Triton X-100 for 20 min. Cells were incubated at room temperature for 20 min with CoraLite^®^594-conjugated Phalloidin antibody (Proteintech Group, Inc, Wuhan, China) at a dilution of 1:100 in the dark. Finally, the slides were sealed with an anti-fluorescence quenching sealer (Servicebio Technology Co., Ltd., Wuhan, China). All the slides were observed, and images were collected using fluorescence microscopy (Thermo Fisher Scientific Co., Ltd, Massachusetts, USA).

Protoscolex at a concentration of 5,000/mL was seeded on glass slides fixed with 4 % paraformaldehyde for 1 h, followed by permeabilization with 0.3 % Triton X-100 for 30 min, and blocked with 4 % goat serum (ZSGB-Bio, Beijing, China) for 1 h at room temperature, and was incubated with anti-Eg.P29 mouse serum or normal mouse serum (1:100) at 4 °C overnight (normal mouse serum as control group). It was then incubated with 1:1000 FITC-conjugated goat anti-mouse IgG antibodies (Abacm, Cambridge, UK) for 1 h. Then the protoscolex of different groups were incubated with phalloidin antibody at a ratio of 1:100 at room temperature for 20 min. Fluorescent signals were detected using fluorescence microscopy.

### 2.11 Detecting mRNA expression of molecules interacting with Eg.P29 by RT-qPCR

Total RNA from HEK293T cells transfected with the Eg.P29 vector or empty vector for 48 h was extracted with TRIzol reagent (Thermo Fisher Scientific Co., Ltd., Massachusetts, USA). mRNA reverse transcription and cDNA synthesis were performed using the First Strand cDNA Synthesis Kit (Thermo Fisher Scientific Co., Ltd., Massachusetts, USA). The mRNA expression of seven genes was detected in different groups using real-time PCR following the instructions for the PowerUpTM SYBRTM Green Master Mix (Thermo Fisher Scientific Co., Ltd., Massachusetts, USA) using a fluorescent quantitative PCR instrument (ABI 7500; Thermo Fisher Scientific Co., Ltd., Massachusetts, USA). The primer sequences are listed in **Supplementary Table S3**. GAPDH was used as a reference gene, and the gene expression level was calculated using the 2^−ΔΔCT^.

### 2.12 Yeast two-hybrid experiment

#### 2.12.1 Construction of bait and prey vectors

The *Eg.P29* sequence was amplified and inserted into pGBKT7 digested by NdeI and BamHI restricted enzyme to construct the bait plasmid *Eg.P29*/pGBKT7. Seven gene sequences (*ACTG1, ACTN4, LIMA1, ARPC1A, VCL, FLNB*, and *MYH10*) were sequentially amplified and cloned into the vector pGADT7 using restriction enzyme cleavage to construct seven different prey plasmids.

#### 2.12.2 Evaluation of toxicity and auto-activation

The Lam/pGBKT7 and T/pGADT7 plasmids were co-transformed into Y2HGold sensory yeast cells as negative controls, and the p53/pGBKT7 and T/pGADT7 plasmids were transformed together as positive controls. The bait plasmid *Eg.P29*/pGBKT7 and empty vector pGADT7 were co-transformed into Y2HGold sensory yeast cells for the self-activation assay. Yeast transformants were coated onto SD/-Trp/-Leu and SD/-Trp/-Leu/-His/-Ade plates. There was colony growth on SD/-Trp/-Leu plates, indicating that the bait plasmid was successfully transferred into the host bacteria and there was no growth on SD/-Trp/-Leu/-His/-Ade plates, indicating that the bait protein was not self-activated. The bait plasmid and empty vector pGBKT7 were transferred into yeast cells and grown on SD/-Trp plates. The growth of the colonies of the bait plasmid was consistent with that of the pGBKT7 empty vector group, indicating that the bait plasmid was non-toxic to yeast cells.

#### 2.12.3 Point-to-point verification of Y2H

The bait plasmid *Eg.P29*/pGBKT7 with the prey plasmids *ACTG1*/pGADT7, *ACTN4*/pGADT7, *ARPC1A*/pGADT7, *FLNB*/pGADT7, *LIMA1*/pGADT7, *MYH10*/pGADT7, or *VCL*/pGADT7 were co-transformed into the Y2H Gold strain, and coated on SD/-Trp/-Leu and SD/-Trp/-Leu/-His/-Ade plates to observe colony growth. The growth of SD/-Trp/-Leu/-His/-Ade indicated a bait and prey protein interaction. To further confirm the authenticity of the validation, the monoclonal dilution spots were picked from above the validation plate and coated on SD/-Trp/-Leu and SD/-Trp/-Leu/-His/-Ade/X-α-gal. When the bait and prey proteins interacted, the yeast colonies turned blue.

### 2.13 Statistical analysis

All statistical analyses were carried out using RStudio software and GraphPad Prism (version 8.4.3). A t-test was used to analyze the continuous data between two groups and one‐way ANOVA was used for three or more sets of data. Results with a *p*-value <0.05 were considered statistically significant. Data are presented as means ± standard deviations.

## 3 Results

### 3.1 IP and LC-MS/MS screening for 50 molecules that interact with Eg.P29 and analyzed through GO and KEGG enrichment

**Figure 1:**
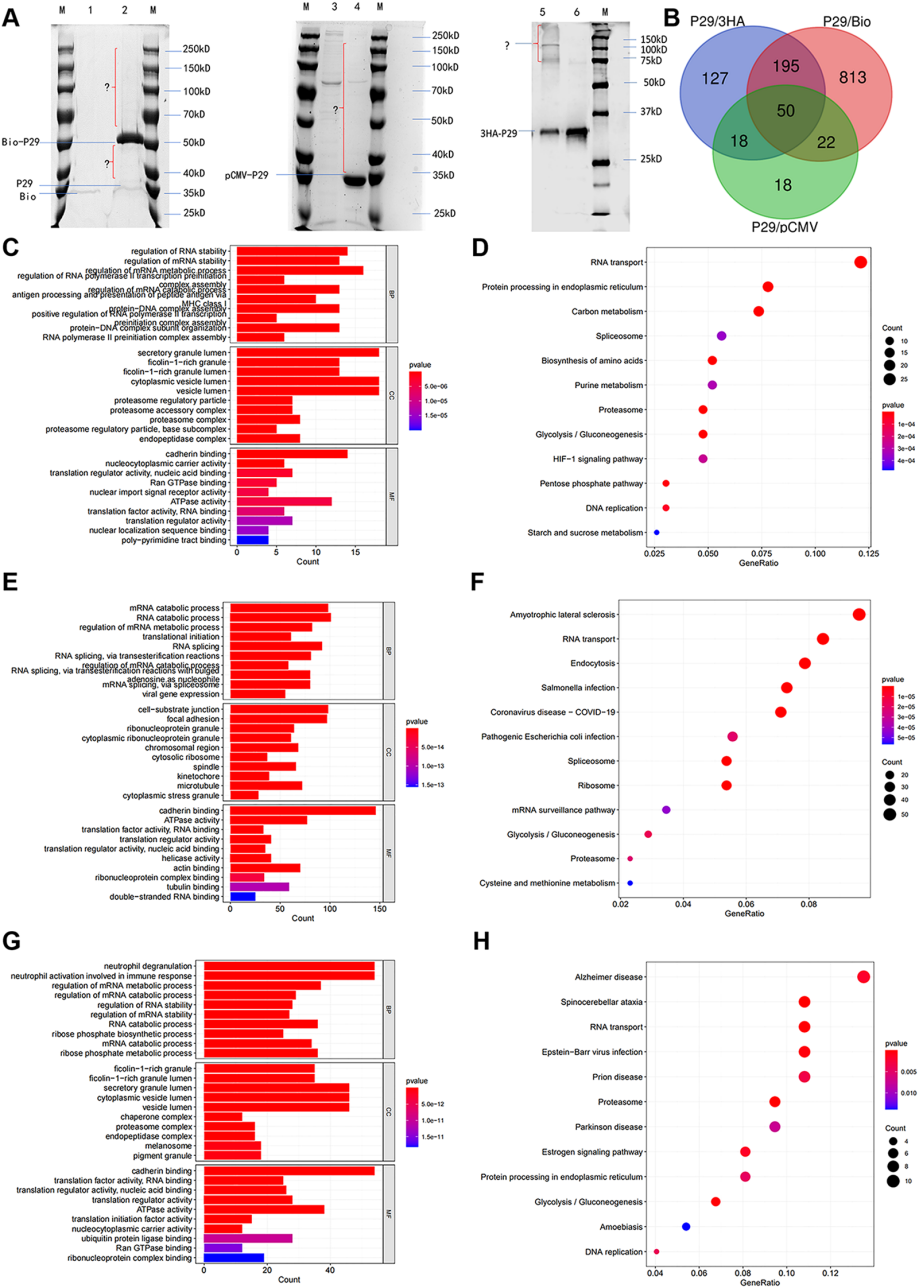
Immunoprecipitation combined with LC-MS/MS screening for 50 molecules that interact with Eg.P29 and analyzed through GO and KEGG enrichment. **(A)** Immunoprecipitation results of three different Eg.P29 vectors. Three different recombinants, namely *P29*/pCMV-4flag, *P29*/3HA-pCDNA3.0, and *P29*/Myc-BioID2-MCS, were constructed and transfected into HEK293T cells, as were the corresponding empty vectors, to obtain protein mixtures through IP methods and separated using SDS-PAGE. The lanes contained the M. Protein molecular weight standard; 1. Myc-BioID2-MCS vector group; 2. *P29*/Myc-BioID2-MCS vector group; 3. pCMV-4flag vector group; 4. *P29*/pCMV-4flag vector group; 5. *P29*/3HA-pCDNA3.0 vector group; 6. 3HA-pCDNA3.0 vector group. **(B)** The diagram of the intersection of LC-MS/MS results of the three IPs. LC-MS/MS analysis of three sets of immunoprecipitated samples acquired differential protein molecules by the two-fold difference in peptide uniqueness (Num Unique) and coverage (%Cov) and these were analyzed against the empty vector set separately. The intersection of the three results was taken to screen for protein molecules that interact with Eg.P29. **(C, D)** GO and KEGG enrichment analysis of differential protein molecules screened in the P29/pCMV-4flag group. **(E, F)** GO and KEGG enrichment analysis of differential protein molecules screened in the *P29*/Myc-BioID2-MCS group. **(G, H)** GO and KEGG enrichment analysis of differential protein molecules screened in the P29/3HA-pCDNA3.0 group. The length of the bar graph indicates the number of genes contained in the pathway, the size of the bubbles in the bubble graph indicates the number of genes contained, and the color indicates the *p*-value.

To find Eg.P29-interacting molecules, three different eukaryotic expression recombinant plasmids were constructed (*P*29/pCMV-4flag, *P*29/3HA-pCDNA3.0, and *P*29/Myc-BioID2-MCS; **Supplementary Figure S2**), as were the corresponding empty vectors, and these were transfected into HEK293T cells to obtain protein mixtures through various IP methods. Visualization was performed using SDS-PAGE. The results show that the three different recombinant vectors of the Eg.P29 groups had different protein bands compared to the corresponding empty vector groups (**Figure 1A**). The IP samples were analyzed using LC-MS/MS. The *Eg.P29*/3HA-pcDNA3.0 recombinant vector group identified a total of 415 proteins, of which 390 were differentially expressed. The *Eg*.*P29*/pCMV-4flag recombinant vector group identified a total of 182 proteins, of which 108 were differentially expressed. The *Eg*.*P29*/Myc-BioID2-MCS recombinant vector group identified a total of 1091 protein molecules, of which 1080 were differentially expressed. For accuracy, the three results were taken as the intersection, and 50 protein molecules that interact with Eg.P29 were screened out (**Figure 1B**). These differentially expressed proteins that interact with Eg.P29 were analyzed for GO and KEGG pathway enrichment. In the *Eg*.*P29*/pCMV-4flag group, the enriched biological functions were mainly cadherin binding, nucleocytoplasmic carrier activity, Ran GTPase binding, and ATPase activity (**Figure 1C**) and the enriched KEGG pathways were mainly RNA transport, spinocerebellar ataxia, glycolysis, and protein processing in the endoplasmic reticulum (**Figure 1D**). In the *Eg.P29*/Myc-BioID2-MCS group, the enriched biological functions were cadherin binding, actin binding, ATPase activity, actin filament-binding, and tubulin binding (**Figure 1E**), and the enriched KEGG pathways were amyotrophic lateral sclerosis, RNA transport, endocytosis, Salmonella infection, and pathogenic *E.coli* infection (**Figure 1F**). In the *Eg.P29*/3HA-pCDNA3.0 group, the enriched biological functions were cadherin binding, ATPase activity, and Ran GTPase binding (**Figure 1G**), and the enriched KEGG pathways were Alzheimer’s disease, protein processing in endoplasmic reticulum, RNA transport, and spinocerebellar ataxia (**Figure 1H**). Cadherin, actin, and small GTPase binding were the main pathways identified.

### 3.2 Eg.P29 expression changes cell morphology and impacts F-actin distribution

**Figure 2:**
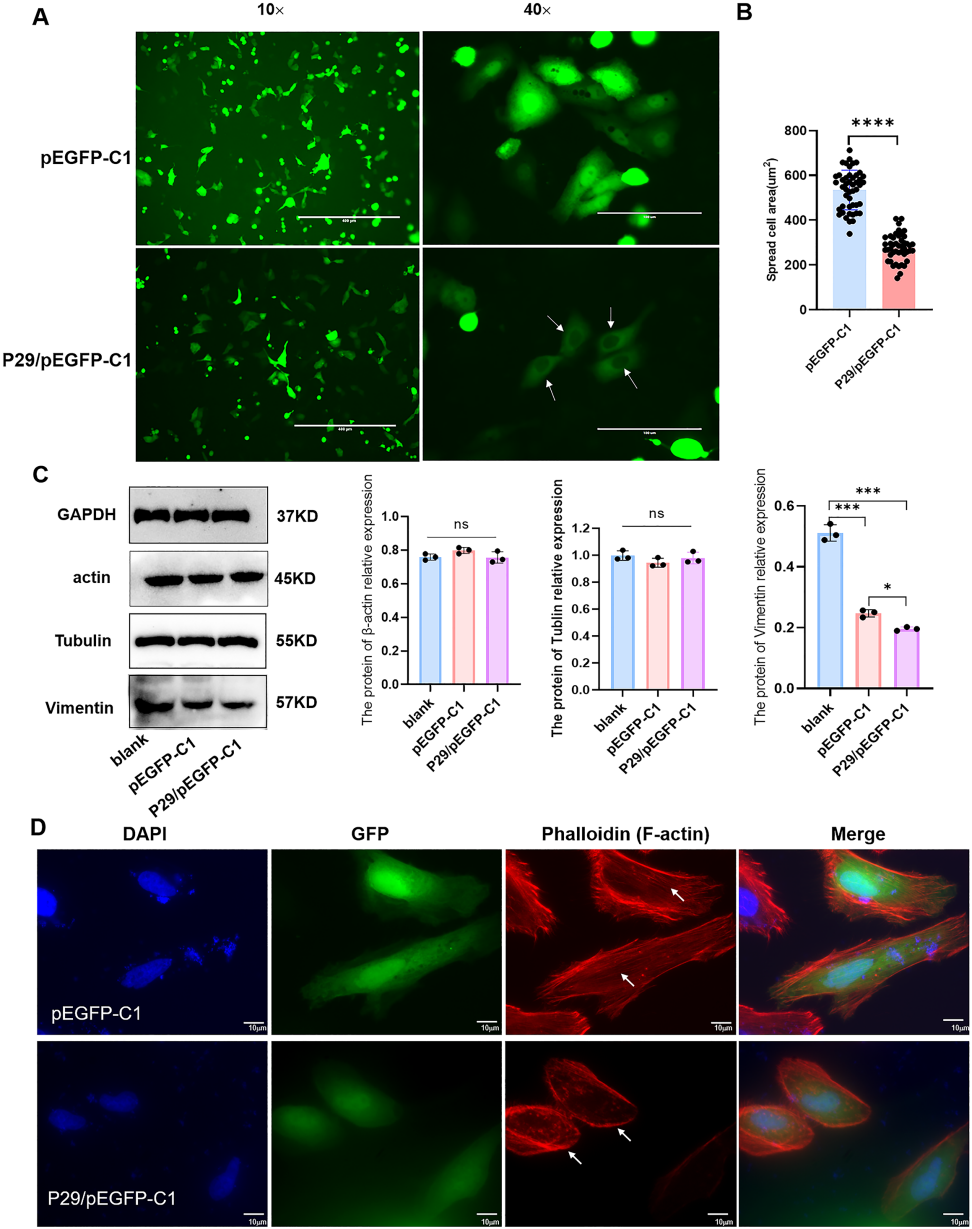
Eg.P29 expression causes cell morphology changes. **(A)** The morphology alteration in cells transfected with Eg.P29 vector. Plasmid *P29*/pEGFP-C1 and empty vector pEGFP-C1 were transfected into HeLa cells for 24h to observe the morphological changes of the cells respectively. The green color represents the cells transfected with the fluorescent expression plasmid and the white arrow shows the morphology alteration of cells. **(B)** Comparison of the average cell area of different groups. The 50 fluorescent transfected cells were randomly selected in both the empty vector group and Eg.P29 vector group and the average cell area of each group was measured. **(C)** The expression of three cytoskeletal proteins in different groups. Cells were collected after being transfected for 24 **(h)** The total protein was obtained by cell lysis buffer. Prepared samples were separated in 10%SDS-PAGE, transferred to PVDF membrane, and recognized with monoclonal antibodies of actin, vimentin, or tubulin by Western blotting. All results are from three independent experiments. Error bars represent standard error of the mean (SEM). ns means no statistical difference, *P<0.05, **P<0.01, and ***P<0.001. **(D)** Plasmid *P29/*pEGFP-C1 and empty vector pEGFP-C1 were transfected into HeLa cells for 24h, respectively. The green color represents cells transfected with the fluorescent expression plasmid and CoraLite^®^594-conjugated Phalloidin antibody labeled the F-actin of cells.

After transfection with the eukaryotic fluorescence-expressing plasmid of *Eg.P29*/pEGFP-C1 and the empty vector pEGFP-C1 into HeLa cells, the cell morphology changed significantly in the Eg.P29 vector group compared with that of the blank and empty groups; the surface of the cells was smooth, round, or oval, without folds and pseudopods, and green fluorescence was only observed in the cytoplasm (**Figure 2A**). The average cell area of the Eg.P29 vector group was significantly smaller than that of the empty vector group (*P*<0.0001) (**Figure 2B**). Three cytoskeletal proteins, tubulin, actin, and vimentin, were detected in different groups (**Figure 2C**). The expression of vimentin in the Eg.P29 vector group was lower than that in the blank group (*P*<0.001) and empty vector group (*P*=0.0332), whereas the expression of actin and tubulin were not statistically significantly different among the groups (*P*=0.2382 and *P*=0.4083, respectively). This indicated that Eg.P29 expression may affected the expression of vimentin. However, in the Eg.P29 vector group, the distribution of F-actin was changed, the stress fibers were reduced and the red fluorescence around the cell membrane was enhanced (**Figure 2D**). This suggested that Eg.P29 may affect actin rearrangement.

### 3.3 Seven actin-related proteins that interact with Eg.P29 were screened out and analyzed

**Figure 3:**
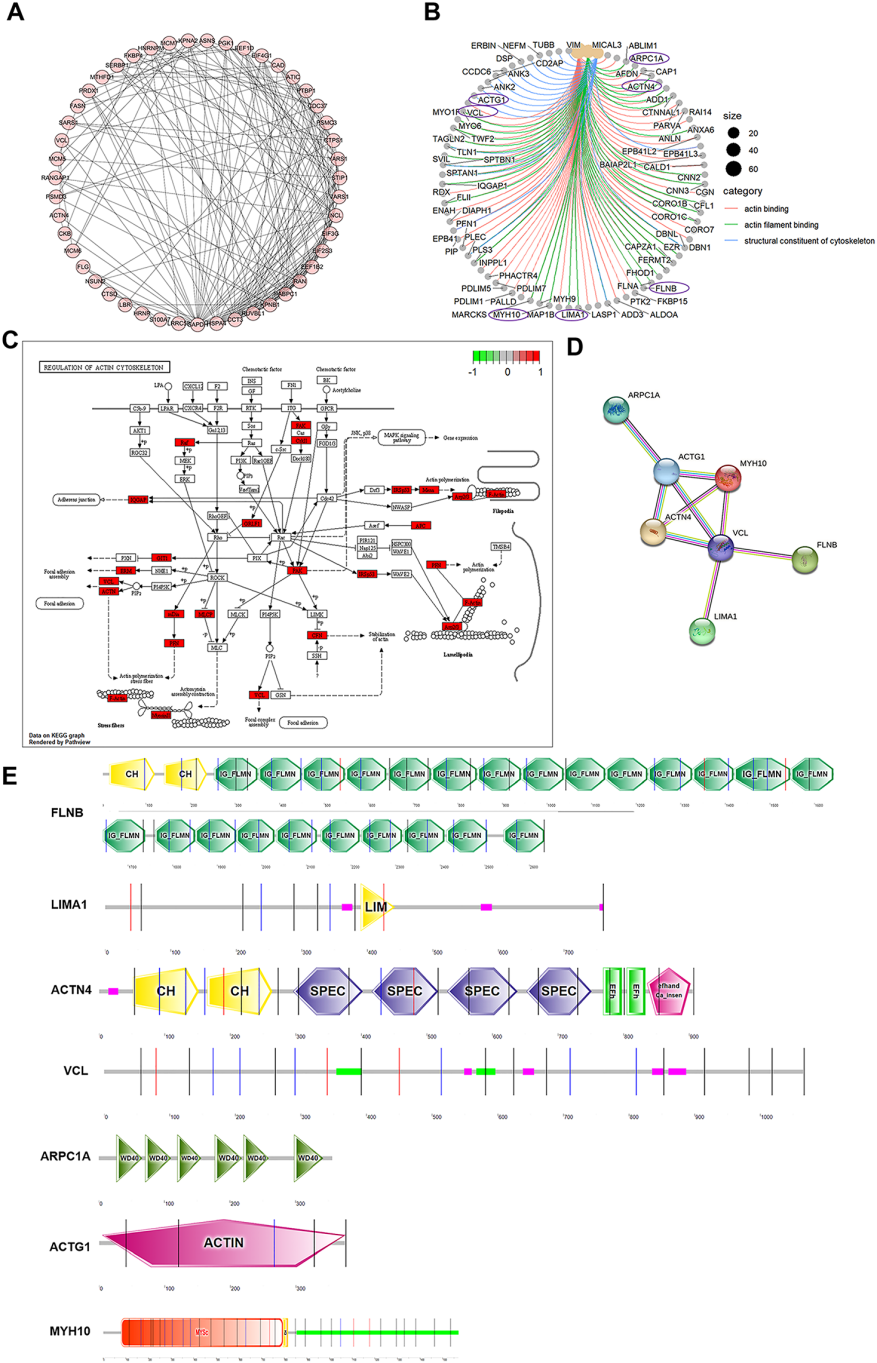
Seven actin-related proteins that interact with Eg.P29 were screened out and analyzed. **(A)** Interactions between 50 molecules that interact with Eg.P29 were analyzed using the STRING database. **(B)** Network map related to actin cytoskeleton regulation obtained from GO analysis. Purple circles mark the seven proteins in the final selection. **(C)** The diagram of the regulation of the actin cytoskeleton. Differential protein molecules from the mass spectrometry results shown to regulate the actin cytoskeleton pathway are highlighted in red. **(D)** Interactions between seven actin-related protein molecules associated with Eg.P29 using the STRING database. **(E)** The prediction diagram of the structural domains of the seven protein molecules using the Prosite database (https://prosite.expasy.org/) and SMART (https://smart.embl.de/).

Because Eg.P29 expression changes the morphology of cells and affects actin distribution, actin-related molecules that interact with Eg.P29 were further screened. First, 50 molecules were analyzed for interactions between them, and 49 proteins comprised 194 edges; the average node degree was 7.92, and PPI enrichment was *P* < 1.0e^-16^ (**Figure 3A**). Next, of the 50 molecules, combined with the results of GO and KEGG enrichment analysis of the actin-related pathway (**Figure 3B**), seven molecules were finally screened out, namely, LIMA1, FLNB, VCL, ACTG1, ARPC1A, MYH10, and ACTN4 and their LC-MS/MS results also showed higher coverage and specificity than the other molecules (**Supplementary Table S5**). Furthermore, ACTN4, VCL, ACTG1, ARPC1A, and MYH10 played key roles in regulating the actin cytoskeleton pathway (**Figure 3C**), and they interacted with each other (**Figure 3D**). The subcellular localization of these molecules was mainly in the cytoplasm, cytoskeleton, and nucleus (**Supplementary Table S6**). Their domains were predicted, showing that both FLNB and ACTN4 contain CH domains, the N-terminal CH domain has the intrinsic ability to bind actin, and FLNB contains a large number of repetitive IG-FLMN domains, which can form a rod-like structure in actin-binding proteins and filamentous proteins. LIMA1 contains LIM domains, which usually bind to protein partners through tyrosine-containing motifs. ARPC1A contains repetitive WD40 domains, a large family of repetitive domains present in all eukaryotes. ACTG1 (actin) mainly contains the ACTIN domain, while MYH10 mainly contains the SH3-like, Myosin-motor, and IQ domains (**Figure 3E**).

### 3.4 Expression changes of seven Eg.P29-interacting molecules

**Figure 4:**
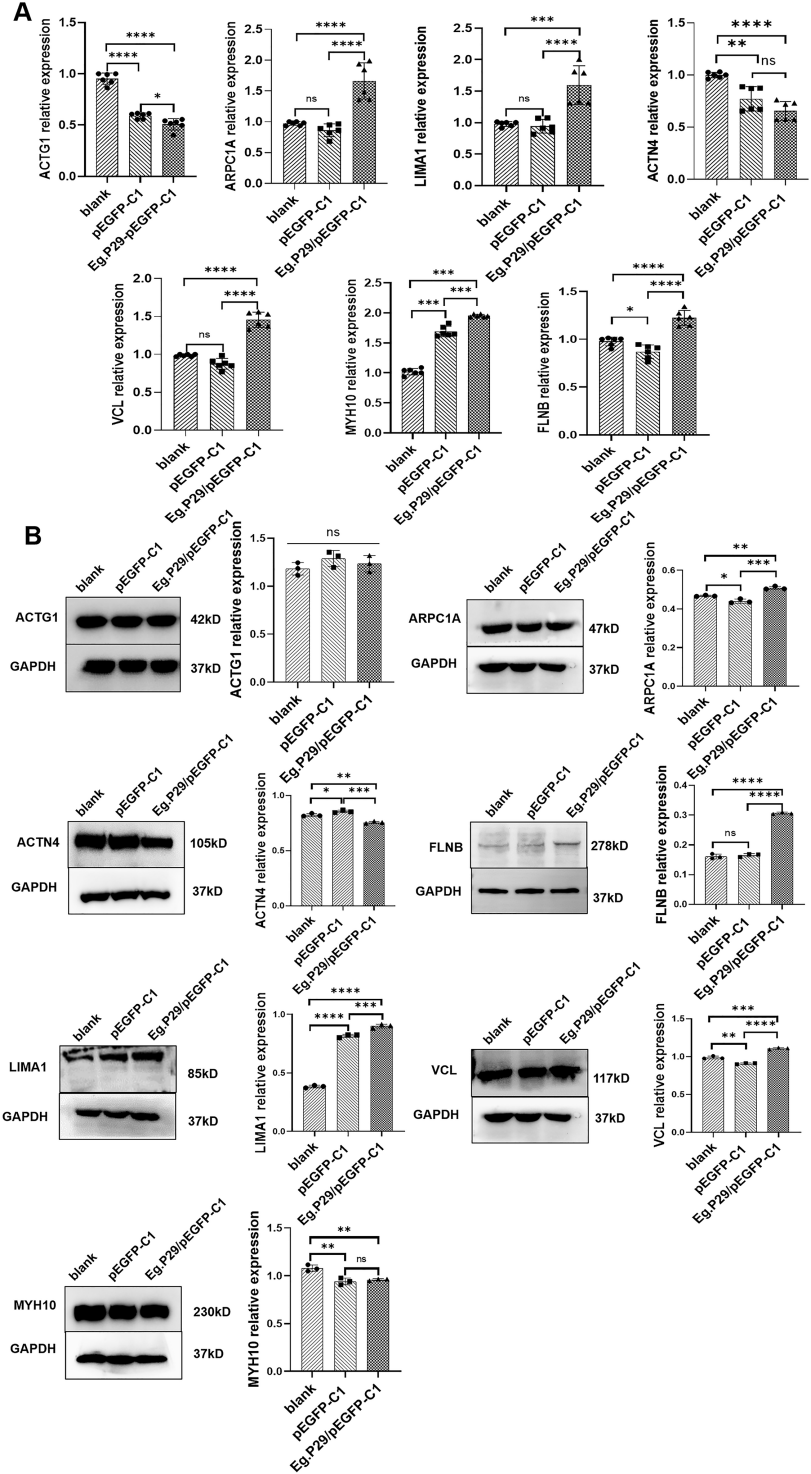
Changes in mRNA and protein expression levels of seven molecules that interact with Eg.P29 in different groups. **(A)** mRNA expression levels of *ARPC1A, LIMA1, FLNB, MYH10, ACTN4, ACTG1*, and *VCL*. HEK-293T cells were transfected with *Eg.P29*/pEGFP-C1vector and the corresponding empty vector for 24 hours. The total RNA was extracted and the mRNA level of specific molecules was shown by PR-qPCR. GAPDH was used as the reference gene, and the 2 ^−δδCt^ method was used to evaluate gene expression. All RT-qPCR experiments were conducted independently six times. Error bars represent the standard error of the mean (SEM). ns means no statistical difference, *P<0.05, **P<0.01, ***P<0.001, and ****P<0.0001. **(B)** The protein expression levels of ACTN4, LIMA1, ARPC1A, FLNB, ACTG1, VCL, and MYH10. HEK-293T cells were transfected with the *Eg.P29*/pEGFP-C1 vector and the corresponding empty vector for 24 hours, and whole cell lysates of different groups were immunoblotted using the specific antibodies. GAPDH was the control protein. ns means no statistical difference, * represents *P*<0.05, ** represents *P*<0.01, *** represents *P*<0.001, and **** represents *P*<0.0001. The results are from three independent experiments. Error bars represent the standard error of the mean (SEM).

The mRNA expression levels of the seven proteins in the Eg.P29 vector, empty vector, and blank control groups were determined using RT-qPCR. Notably, the expression of *ACTN4* and *ACTG1* were significantly lower in the Eg.P29 vector group than in the blank control group (*P*<0.0001) and empty vector group (*P*=0.0980) (*P*<0.05). Conversely, the expression of *LIMA1, FLNB, ARPC1A*, *MYH10*, and *VCL* were all higher in the Eg.P29 vector group than in the blank control group (*P*<0.001) and empty vector group (*P*<0.001), and there was no statistically significant difference between the expression of these molecules in the blank control group and empty vector group. (**Figure 4A**). The protein expression levels of ARPC1A, LIMA1, FLNB, and VCL were upregulated by Eg.P29, whereas the expression levels of ACTN4 and MYH10 were slightly downregulated with Eg.P29, and the expression of ACTG1 was not statistically significantly different among the groups (**Figure 4B**).

### 3.5 Verification of the Eg.P29-interacting molecules LIMA1 and actin using a yeast two-hybrid experiment and Co-IP

**Figure 5:**
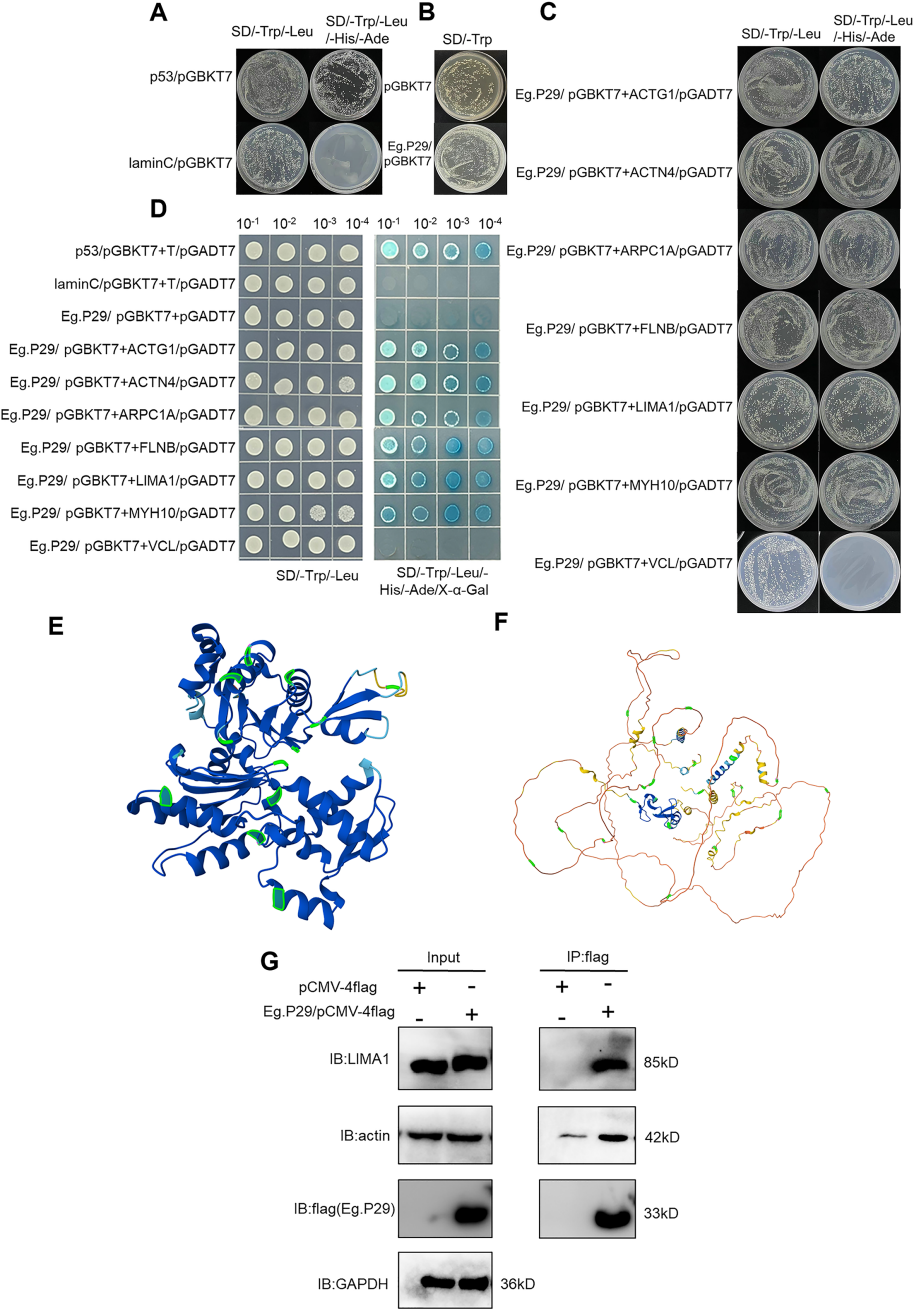
Verification of Eg.P29-interacting molecules LIMA1 and actin by yeast two-hybrid experiment and co-immunoprecipitation. **(A)** The self-activation assay of Eg.P29. The bait plasmid *Eg.P29*/pGBKT7 and the empty vector pGADT7 were co-transformed into Y2HGold sensory yeast cells for the self-activation assay. The Lam/pGBKT7-T/pGADT7 plasmids were used as a negative control, and the p53/pGBKT7-T/pGADT7 plasmids were used as a positive control. **(B)** Toxicity assay of Eg.P29. We compared colony growth transformed with the pGBKT7 empty vector and colonies transformed with the *Eg.P29*/pGBKT7 vector on an SD/-Trp plate for the toxicity assay. **(C)** Point-to-point validation of interaction molecules with Eg.P29. The bait plasmid *Eg.P29*/pGBKT7 and seven different prey plasmids were co-transformed into Y2HGold yeast cells and coated on SD/-Trp/-Leu/-His/-Ade plates. The interaction relationship of the bait protein and prey proteins was determined by the colony’s growth or not on plates. **(D)** Spot plate validation of interaction molecules. Selected monoclonal dilutions were cultured on an SD/Leu/-Trp/-His/-Ade/X-α-gal plate for spot plate validation to confirm the interaction of seven protein molecules with Eg.P29 based on whether the colonies’ color changed to blue. **(E)** Localization of the homologous regions of ACTG1, FLNB, and ARPC1A in the spatial structure of ACTG1. Sequence comparison was performed using MEGA software, the ACTG1 model structure was queried, and homology regions were labeled using the AlphaFold Protein Structure Database. The homologous regions were labeled in green in the figure. **(F)** Localization of the homologous regions of LIMA1, ACTN4, and MYH10 in the spatial structure of LIMA1. The homologous regions were labeled in green in the figure. Sequence comparison and modeling are the same as **(E)**. **(G)** Eg.P29/pCMV-4flag plasmid was transfected into HEK293T cells, and cell lysates were mixed with Anti-flag magnetic beads and then eluted with FLAG peptide to collect the precipitation complexes using a primary antibody to detect interaction molecules by western-blot. GAPDH was the intrinsic reference protein. These experiments were repeated three times.

Growth and color changes in the hybrid yeast allowed for the evaluation of interactions between Eg.P29 and the seven protein molecules. No colonies grew on the SD/-Trp/-Leu/-His/-Ade and SD/-Trp plates, indicating that the bait protein Eg.P29 had no self-activation (**Figure 5A**) and was non-toxic to the yeast cells, similar to the empty vector (**Figure 5B**). The bait plasmid *Eg.P29*/pGBKT7 and the prey plasmids *ACTG1*/pGADT7, *ACTN4*/pGADT7, *ARPC1A/*pGADT7, *FLNB*/pGADT7, *LIMA1*/pGADT7, and *MYH10*/pGADT7 were constructed (**Supplementary Figure S3**) and co-transformed respectively to yeast cells and grew well on SD/-Trp/-Leu/-His/-Ade plates the same as the positive control *p53*/pGBKT7. This suggested that the bait proteins of Eg.P29 interact with prey proteins ACTG1, ACTN4, ARPC1A, FLNB, LIMA1, and MYH10 (**Figure 5C**). In addition, the yeast of six molecules showed blue colonies when cultured on an SD/-Leu/-Trp/-His/-Ade/X-α-gal plate. This indicated that Eg.P29 was a one-to-one interaction between ACTG1, ACTN4, ARPC1A, FLNB, LIMA1, and MYH10 molecules. However, in the VCL group, the plate failed to produce results similar to those observed in the other molecular groups, indicating the absence of an interaction between Eg.P29 and VCL (**Figure 5D**). The homology of the six proteins were compared, except for VCL, and we found that the six proteins can be roughly categorized into two groups of proteins led by LIMA1 and ACTG1, respectively. The members of the two groups are ACTN4 and MYH10, and FLNB and ARPC1A, respectively (**Supplementary Table S8**). The homology regions of the three member proteins of the two groups were compared (**Supplementary Figures S4, S5**) and labeled on the model structures of LIMA1 and ACTG1, respectively (**Figures 5E, F**).

Finally, the interaction of Eg.P29 with ACTG1, ACTN4, ARPC1A, FLNB, LIMA1, and MYH10 was identified using a Co-IP assay. The results showed that actin and LIMA1 molecules were detected in protein complexes with flag labeling in Eg.P29-transfected HEK293T cells, indicating that LIMA1 and actin interacted with Eg.P29 (**Figure 5G**).

### 3.6 P29 and actin were co-localized on the rostellum and suckers of the protoscolex

**Figure 6:**
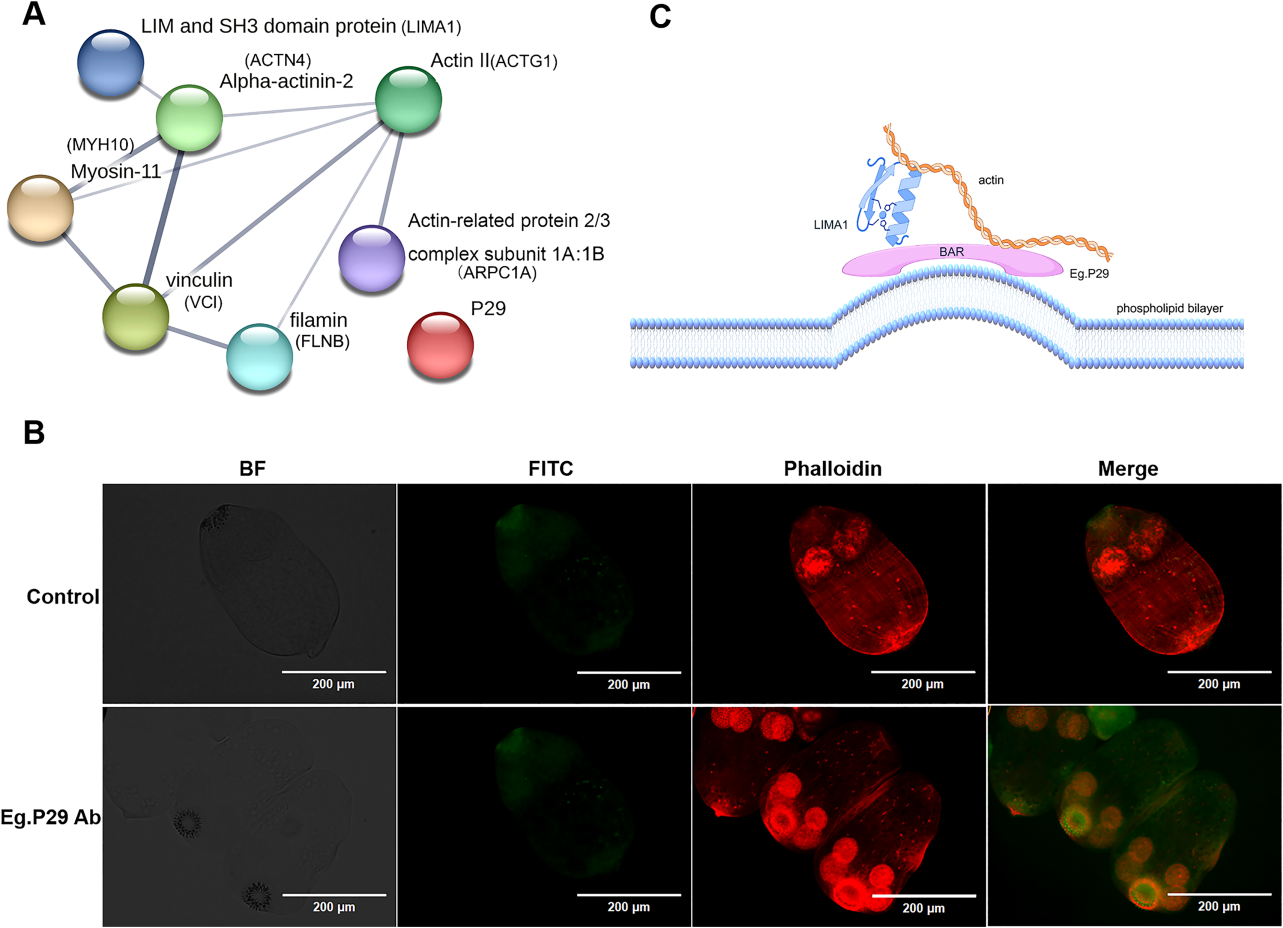
Prediction of the relationship between seven homologous protein molecules that interact with Eg.P29 in (*E*) *granulosus* and detection of the protein expression on the protoscolex. **(A)** Homologous molecules interacting with Eg.P29 found in *E.granulosus*. Using the NCBI database BLAST, seven protein molecules from human cells were sequentially searched for the protein with the highest homology in (*E*) *granulosus*. The homologous proteins create an interaction network through the STRING database. **(B)** Co-location of P29 and actin on the protoscolex. Protoscolices coated on a glass slide were incubated with normal mouse serum or anti-Eg.P29 mouse serum overnight, incubated with FITC-conjugated goat anti-mouse IgG antibody, and then incubated with CoraLite^®^594-conjugated Phalloidin antibody at room temperature. Normal mouse serum as control serum. Phalloidin antibody shows the F-actin distribution. FITC-conjugated goat anti-mouse IgG antibody shows the P29 distribution. **(C)** Mimic relationship diagram of P29, LIMA1, and actin (Figdraw).

To further identify protein molecules that interact with P29 in *E. granulosus*, we discovered homologous molecules corresponding to seven human proteins within *E. granulosus*, and all of them retained the same domains as their human homologous proteins, except for Eg.LIMA1, which includes an additional SH3 domain compared to LIMA1 in humans. Notably, the homology between Eg.actin and human actin protein was 96.26 %, with ACTN4 (54.45 %), ARPC1A (50.31 %), MYH10 (50.23 %), LIMA1 (41.48 %), FLNB (38.69 %), and VCL (33.80 %) following closely behind (**Supplementary Table S7**). These seven molecules from *E. granulosus* formed intricate protein interaction networks in the prediction (**Figure 6A**). Furthermore, the precise localization of P29 and actin within the protoscolex has been unveiled through tissue immunofluorescence. The findings reveal P29 and actin were expressed in both the rostellum and the suckers of the protoscolex (**Figure 6B**).

## 4 Discussion

Eg.P29 plays a crucial role in the growth and development of the *E. granulosus* metacestode. However, the intricate life cycle and developmental processes of *E. granulosus* pose significant challenges for researchers. Currently, there is a dearth of pertinent reports concerning the function of Eg.P29.

### 4.1 Properties and function of Eg.P29 predicted using a bioinformatics database

Initially, we employed a diverse array of bioinformatics database resources to prospectively dissect the fundamental attributes, structure, and functional characteristics of Eg.P29. The findings revealed that the Eg.P29 protein possessed good stability (**Supplementary Table S4**) and was soluble in aqueous environments (**Supplementary Figure S1A**). This observation aligns with the outcomes of previous experiments that successfully isolated soluble Eg.P29 proteins *in vitro* (Shi et al., 2009). Due to the absence of transmembrane domains and signal peptides in Eg.P29 (**Supplementary Figures S1B, C**), it does not qualify as a secreted protein or transmembrane protein (González et al., 2000). Instead, it resides in the cytoplasm, which is aligned with the predicted subcellular localization results (**Supplementary Figure S1D**). Determining the location of Eg.P29 provides key information for exploring the functions and roles played by the Eg.P29 protein in cells.

Furthermore, Eg.P29 homologous proteins were identified using the BLAST algorithm from the NCBI, and the amino acid sequence of Eg.P29 was demonstrated to have 100 % sequence homology with endophilin B1 found in *E. granulosus* (**Figure 2B**). In humans, Endophilin B1 is a protein containing SH3 and BAR domains, which are involved in membrane remodeling and vesicle formation (Li et al., 2016). It is also essential for synaptic vesicle transmission (Modregger et al., 2002), and the inhibition of its expression leads to defective synaptic vesicle endocytosis (Schuske et al., 2003). Therefore, we hypothesized that the Eg.P29 (Eg.endophilinB1) protein may have a function similar to that of Endophilin B1 in humans. Structural domain analysis revealed that Eg.P29 is indeed a protein that contains an N-BAR domain (**Supplementary Figure S1E**). Most proteins containing BAR domains are encoded by eukaryotic genomes, and they exist universally, spanning from unicellular eukaryotes, such as yeast, to complex organisms (Ren et al., 2006). These proteins exhibit exceptional versatility in their involvement in various cellular processes, including synaptic vesicle fission, cell polarity, endocytosis, actin cytoskeleton regulation, and transcriptional repression (Carman and Dominguez, 2018). Proteins with an N-BAR domain have a high propensity to adopt an α-helical structure (**Supplementary Figure S1G**) and engage in coiled-coil interactions with other proteins (Habermann, 2004; Ren et al., 2006), ultimately leading to alterations in the membrane curvature (Mim and Unger, 2012; Pang et al., 2014). Therefore, Eg.P29, as a member of the BAR domain protein family, is predicted to play a major role in the establishment or maintenance of actin polarity and endocytosis as other BAR domain proteins do (**Supplementary Figure S1F**).

### 4.2 Screening actin-related molecules of interaction with Eg.P29

Based on the above information, we further explored the role of P29 in *E. granulosus.* However, there are few reports on the functions of the protein molecules in *E. granulosus* due to the absence of an appropriate model organism for this large parasite. Furthermore, proteins expressed *in vitro* exhibit limited activity and fail to mirror their functional capabilities in an organism. Therefore, identifying suitable research hosts is imperative.

According to the literature, Liu et al (Liu et al., 2017). found that Ravk could disrupt the host cytoskeleton structure by cleaving actin during Legionellosis using 293T cells. Kumar and Valdivia (2008) studied the expression of cytoskeletal proteins in HeLa cells infected with *Chlamydia trachomatis*, and Jabari et al. (2018) compared the culture of *Toxoplasma gondii* in four cell lines, HeLa, Vero, RBK, and A549, and found that the HeLa and Vero cell lines were suitable for the rapid and long-term propagation of *Toxoplasma gondii*, respectively. These studies underscored the potential of employing human cell lines to explore Eg.P29-interacting molecules, which is essential for further research. IP combined with LC-MS/MS has become an effective technique for the detection of protein interactions (Moresco et al., 2010; Seger and Salzmann, 2020); it can rapidly and efficiently detect a large number of proteins and is extremely important for revealing the functions and relationships of unknown proteins in cells (Santos Seckler et al., 2021). BioID is a novel neighborhood-dependent labeling technique for proteins in eukaryotic cells that can be used to detect weak and transient interactions between proteins (Roux et al., 2012; Roux et al, 2018) and may complement IP. Therefore, we constructed the eukaryotic recombinant plasmids *Eg.P29*/Myc-BioID2-MCS, *Eg.P29*/pCMV-4flag, and *Eg.P29*/pCDNA3.0-3HA (**Supplementary Figure S2**), transfected them into HEK293T cells, and screened for the Eg.P29-interacting molecules using IP combined with LC-MS/MS (**Figure 1A**). To improve the screening rate, 50 molecules were obtained by intersecting three sets of LC-MS/MS results (**Figure 1B**). Additionally, we constructed the eukaryotic fluorescent recombinant plasmid *Eg.P29*/pEGFP-C1 that was transfected to Hela cells. The results revealed that the cellular morphology in the Eg.P29 vector group became smaller and a round or elliptical shape, with a notable disappearance of pseudopods on their surface in comparison with other groups (**Figures 2A, B**). It is speculated that Eg.P29 expression may influence cytoskeletal formation. The alterations in cytoskeleton morphology mainly involved microtubules, microfilaments, and intermediate filaments. Moreover, protein expression of tubulin, actin, and vimentin was detected in different groups, showing that Eg.P29 inhibited vimentin expression (**Figure 2C**). However, vimentin belongs to a class of intermediate filament proteins that exhibit remarkable tissue specificity, with distinct intermediate filament proteins present in different cell types (Battaglia et al., 2018). Furthermore, vimentin expression was also notably reduced in the empty vector group, suggesting that the pEGFP-C1 vector affected vimentin expression levels. In contrast, actin is highly conserved and expressed in most eukaryotic cells, has no tissue specificity (Dominguez and Holmes, 2011), is mainly distributed near the cell membrane, and is used to form stress fibers (Pollard and Cooper, 2009). We found the distribution of F-actin was changed, accumulating under the cell membrane (**Figure 2D**). Therefore, we hypothesize that the changes in cell morphology were attributable to actin rearrangement (Tsarfaty et al., 2000). Additionally, the results of the LC-MS/MS indicated that actin and actin-related proteins had a very high coverage and specificity. It is speculated that Eg.P29 may interact with actin. Actin is a common target of many intracellular and extracellular bacterial and pathogen research studies (Javier-Reyna et al., 2023). To further explore the relationship between Eg.P29 and actin, we focused on screening actin and associated proteins that interact with Eg.P29. Ultimately, seven key molecules, ACTN4, VCL, ACTG1, LIMA1, FLNB, ARPC1A, and MYH10 were screened out (as depicted in **Figures 3B, C**), and these formed a complex interaction network (**Figure 3D**).

### 4.3 Validation and identification of interaction molecules with Eg.p29

The mRNA and protein expression levels of the seven molecules in different groups were validated by RT-qPCR and Western blotting, respectively, to yield a preliminary understanding of their response to Eg.P29 expression.

ACTG1 encodes γ-actin. β and γ actin coexist in most cell types as components of the cytoskeleton and mediators of internal cellular motility (Arora et al., 2023). β and γ actin were found to have 91.7 % homology; thus, actin expression can be characterized by ACTG1 expression in cells. In our study, Eg.P29 did not impact ACTG1 expression. ACTN4 contains the CH domain, which can bind to actin or various cytoskeleton molecules (Stradal et al., 1998) involved in the structural organization of the cytoskeleton (Tentler et al., 2019) (**Figure 4**). However, FLNB exhibited higher expression levels in the Eg.P29 vector group than in the other groups, which is consistent with the findings of Hu (Hu et al., 2017). The prolonged loss of FLNB promotes the formation of actin stress fibers, negatively regulating actin expression in cells. As a constituent of the filamin protein family, FLNB plays a pivotal role in regulating actin dynamics and facilitating vesicular transport (Gorlin et al., 1990; Cunningham et al., 1992). Except for MYH10, LIMA1, ARPC1A, and VCL showed similar results to those of FLNB. LIMA1 plays a pivotal role in regulating actin dynamics and structure and promoting the quantity and length of stress fibers. It effectively hinders the depolymerization of actin filaments and facilitates their crosslinking into organized bundles (Maul et al., 2003). ARPC1A is associated with the control of actin polymerization in cells and mediates the formation of branched actin networks along with the activation of nucleation-promoting factors (Balcer et al., 2010). VCL is a major component of adhesion plaques and plays a key role in cytoskeletal remodeling and signal transduction (Wu et al., 2024). Thereby, Eg.P29 may affect these molecules’ expression to regulate cytoskeleton remodeling. MYH10 belongs to traditional non-muscle myosin and, as an actin-dependent motor, has multiple functions including regulating cytoplasmic division, cell movement, and cell polarity (Takagi et al., 2014). Despite demonstrating heightened levels of both mRNA and protein expression within the Eg.P29 vector group compared to the blank group, the results of mRNA and protein were conflicting, and no statistically significant differences were discernible with the empty vector group; therefore, the expression alteration was not attributable to the influence of Eg.P29. Yeast two-hybrids are commonly used to identify direct interactions between proteins (Serebriiskii and Kotova, 2004; Brückner et al., 2009). A thorough investigation of the interaction relationships between several molecules and Eg.P29 revealed that all molecules except VCL exhibited direct interactions with Eg.P29 (**Figures 5A–D**). However, upon thorough analysis, we acknowledge that this finding carries a significant level of uncertainty, as it may stem from the occurrence of homologous sequences shared among these molecules or the potential formation of large protein complexes, which could result in spurious positive outcomes in reporter gene expression assays (Rodríguez-Negrete et al., 2014). Homology sequence alignment of the six molecules revealed that ACTG1 displayed a homology exceeding 42 % with both FLNB and ARPC1A, whereas LIMA1 exhibited a homology greater than 45 % with ACTN4 and MYH10 (**Figures 5E, F**). To enhance the precision of our findings, Co-IP was performed for identification, conclusively verifying the interaction between Eg.P29 and both LIMA1 and actin (**Figure 5G**). Interestingly, Maul et al Maul et al., 2003). confirmed that LIMA1 directly engages with actin. Consequently, Eg.P29, LIMA1, and actin interact to form a compound regulatory system that modulates the expression of downstream molecules, such as ARPC1A, ACTN4, VCL, and FLNB (Cunningham et al., 1992; Wang et al., 2023). However, the seven molecules are all human proteins. Does P29 also engage in interactions with these molecules within *E. granulosus*? Upon investigation, we pinpointed seven homologous molecules to the human proteins in *E. granulosus*, which formed an intricate network of interacting proteins centered on actin (**Figure 6A**). Notably, the *Eg.actin* exhibits a high degree of homology with human *actin*, reaching 96.26 % (da Silva et al., 1993). Therefore, we are fully convinced that P29 interacts with actin in *E.granulosus*. Furthermore, the remarkable co-localization of P29 and actin in both the rostellum and the sucker of the protoscolex reinforces this hypothesis (**Figure 6B**). The result was consistent with the studies by Gonzalez et al., Zhang et al., and Silvana et al. on the location of P29 (González et al., 2000; Zhang et al., 2024) and actin (La-Rocca et al., 2019) in *E. granulosus*. They may collectively determine the direction of movement, the energy requirements necessary for adaptation to the environment, and the function of the rostellum and suckers (La-Rocca et al., 2019).

However, to elucidate the precise role of P29 in *E. granulosus*, a series of questions must be addressed, such as whether Eg.P29 also interacts with Eg.LIMIA1, and if the complex interaction relationship among Eg.P29, Eg.LIMIA1, and Eg.actin persists, which necessitates more rigorous and exhaustive investigations.

In summary, our study revealed that Eg.P29 is a soluble protein located in the cytoplasm that is capable of influencing the expression of actin-related molecules. Eg.P29 was confirmed to interact with LIMAI and actin, forming a compound regulation system to affect cytoskeleton formation, as shown in the mimics diagram (**Figure 6C**). We also deduced that Eg.P29 interacts with Eg.actin in *E. granulosus*. This study has established a crucial foundation for investigating the functionality of P29. Nevertheless, the manner in which the interplay between P29 and actin impacts the development and activity of the protoscolex and its regulation of specific mechanisms involving other molecules remains unclear and necessitates further in-depth studies.

## Data Availability

The original contributions presented in the study are included in the article/**Supplementary Material**. Further inquiries can be directed to the corresponding author.
