# Supplementary material for: Screening and identification of protein 29 of Echinococcus granulosus interacting molecules
Source: Front Cell Infect Microbiol. 2025 May 2;15:1560436. doi: 10.3389/fcimb.2025.1560436 (PMC12081405; doi:10.3389/fcimb.2025.1560436)
Supplement: Supplementary file 1 [file DataSheet1.pdf]

Supplementary Material

1 Supplementary Materials and methods

1.1 Prediction of the basic properties, structure, and function of Eg.P29

The Eg.P29 amino acid sequence in fasta format was submitted directly to all of the following online databases for the prediction of results. Various physical and chemical parameters of Eg.P29, including the molecular weight, theoretical isoelectric point, amino acid composition, atomic composition, extinction coefficient, half-life, and instability index, were predicted using the ProtParam tool on the online prediction site SIB Expasy(https://www.expasy.org/). The hydrophobicity of Eg.P29 was predicted using the ProtScale website, the Hphod./Hoop &Woods algorithm was selected, and the sliding window size was set to 19 to avoid stray peaks. Protein–sol, a database was used to predict the solubility of Eg.P29. Amino acid sequences are entered into the software for comparison with the solubility database and a set of solubility prediction calculations are returned. If the return value greater than average solubility of 0.45 was considered soluble (Maryam et al., 2024). The DeepTMHMM database was used to analyze the transmembrane region of Eg.P29, and SignalP6.0 was used to predict the presence of a signal peptide in Eg.P29. The subcellular localization of Eg.P29 was predicted using Cell-PLoc 2.0 database. Select the Euk-mPLoc 2.0 server in which the subcellular localization of eukaryotic proteins can be predicted. Moreover, the Eg.P29 domain was predicted using Prosite and validated using SMART. The PredictProtein database predicted the functions of Eg.P29, including the biological processes, components, and functions involved. The secondary structure of Eg.P29 was predicted by using PSIPRED database to understand the proportion of α-helix and β-folding in the sequence of Eg.P29. The tertiary structure of Eg.P29 was simulated using the Phyre2 database. The specific Uniform Resource Locators (URLs) are presented in Table S1.

1.2 Homology analysis

The search for Eg.P29 homologous sequences by BLAST algorithm at the National Center for Biotechnology Information (NCBI: https://blast.ncbi.nlm.nih.gov/) , and the default non-redundant protein sequence (nr) database was selected after inputting the Eg.P29 protein sequence in the BLAST program to screen the top 20 species with the highest homology for multi-sequence comparisons. Phylogenetic tree was constructed using the MUSCLE algorithm in MEGA11. The Neighbor-Joining Method (NJ) was used for analysis, the Jukes-Cantor distance was calculated using this method to determine the rationality of constructing a tree. The tree was reconstructed 1000 times and beautified using the evolview website (https://evolgenius.info//evolview-v2). The six parasite species with the highest homology to Eg.P29 in the phylogenetic tree were selected for detailed amino acid sequence comparison and domain analysis and visualized using the ESPript3 website (https:// espript.ibcp.fr/ESPript/ESPript/).

2 Supplementary Results

2.1 Soluble Eg.P29 that exists in the cytoplasm contains N-BAR domain

Eg.P29 consists of 238 amino acids, including 41 acidic amino acids and 37 alkaline amino acids, with a molecular weight of 27096.59, theoretical isoelectric point of 5.63, and half-life of 30 h (Table S4). It is a stable, hydrophilic protein, as predicted by the ProtScale database (Figure S1A). This protein is absent in the transmembrane region (Figure S1B) and signal peptide (Figure S1C). These data indicate

that Eg.P29 is a highly conserved and non-secretory protein that exists in the cytoplasm (Figure S1D). Based on the sequence information, Eg.P29 has a large domain from position to 5-238 called the BAR domain (Figure S1E). The biological functions of Eg.P29 may be involved in endocytosis and establishment or maintenance of actin cytoskeleton polarity and is involved in the biological components of the Golgi membrane, Golgi apparatus, cytoplasm, and cytoskeletal protein binding (Gang et al., 2006) (Figure S1F). The proportion of α-helices was very high (86.97%), irregular coils accounted for 12.18%, and β-folding accounted for 0.42% (Figure S1G), indicating that the structure of Eg.P29 was stable. The tertiary structure prediction model showed a crescent shape similar to that of the N-BAR domain (Figure S1H).

2.2 Eg.P29 has higher homology to some parasites

A phylogenetic tree was constructed using MEGA11 software to analyze the evolutionary process of Eg.P29. The results showed that Eg.P29 sequences were closely related to the sequences of *E. multilocularis*, *Echinococcus equinus*, and *Echinococcus canadensis* (Figure 1I), which are all parasite sequences. The top six sequences (P29 of *E. multilocularis*, P29 of *E. equinus*, P29 of *E. canadensis*, antigen 6 of *E. multilocularis*, unnamed protein of *Taenia asiatica*, and P27 of *Taenia solium*) with Eg.P29 for amino acid sequence alignment revealed that Eg.P29 (EndophilinB1 of *E.granulosus*) has high homology with these sequences, ranging from 79.4% to 98.7%, and they all contain the BAR domain, as shown by the red dashed line region. Most have the same amino acid (shown in yellow in Figure 1J), which indicates that P29 is a highly conserved protein among different parasite species.

3 Supplementary Figures and Tables

3.1 Supplementary Figures

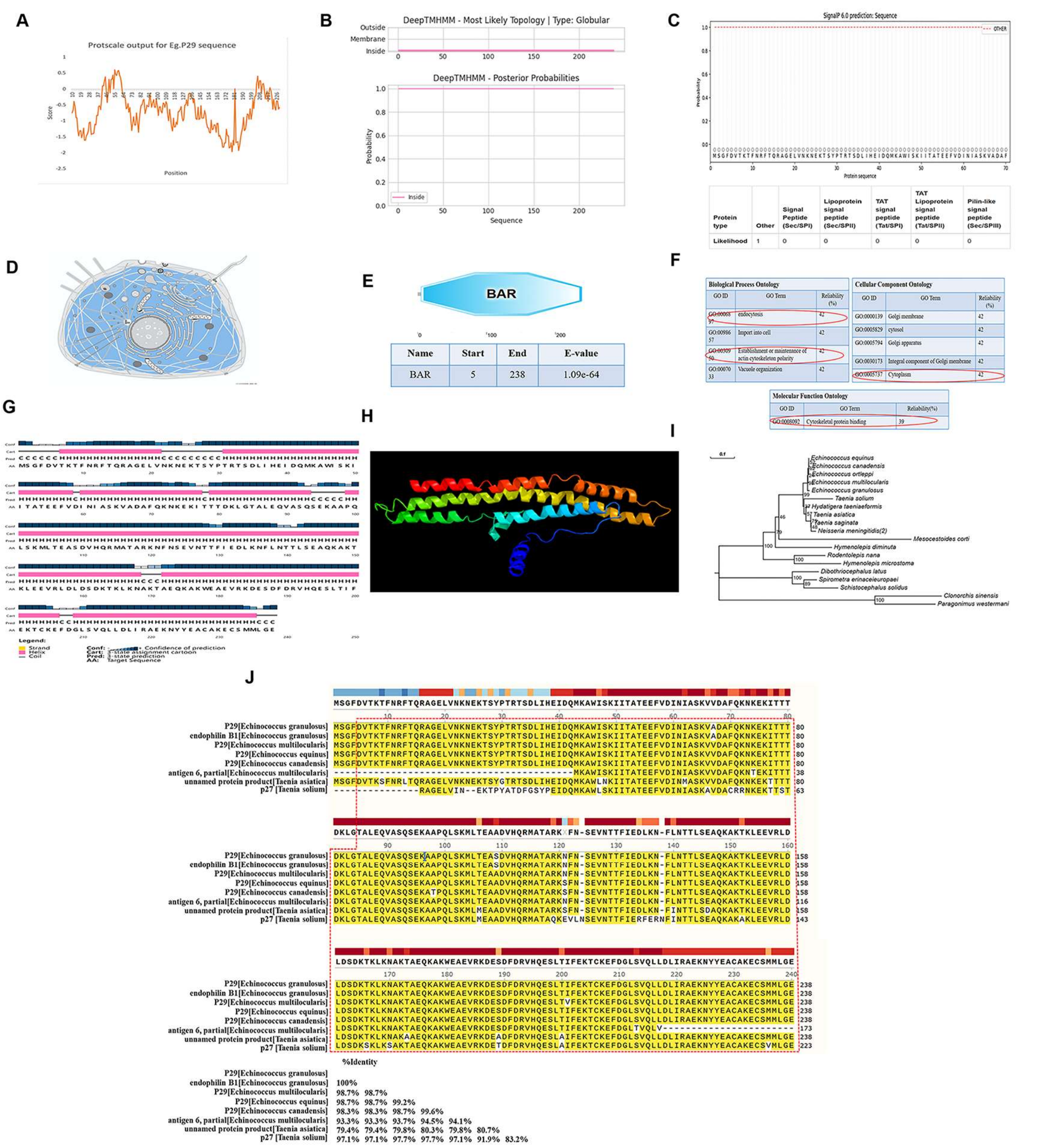

Supplementary Figure 1. Soluble Eg.P29 that exists in the cytoplasm contains N-BAR domain and has higher homology to some parasites

(A) The diagram of Eg.P29 hydrophilicity prediction. Hydrophilicity/ hydrophobicity of Eg.P29 were predicted by ProtScale database, the GRAVY value of is -0.697<0; (B)The diagram of transmembrane prediction of Eg.P29 with DeepTMHMM database; (C) The diagram of signal peptide prediction of Eg.P29 by SignalP6.0 database; (D) The diagram of subcellular localization prediction of Eg.P29 with Cell-PLoc 2.0 database, the blue part indicates the region where Eg.P29 exists; (E) The domain of Eg.P29 predicted by SMART(https://smart.embl.de/); (F) Biological functions of Eg.P29 predicted by PredictProtein database, red circle shown the results; (G) Secondary structure Eg.P29 predicted by PSIPRED database， pink color represent α-helices; (H) Tertiary structure of Eg.P29 simulated by Phyre2 database (http://www.sbg.bio.ic.ac.uk/phyre2/html/page.cgi?id=index); (I) The diagram of phylogenetic tree of Eg.P29. The top 20 different species with the highest homology were screened for multi-sequence comparisons and constructed a phylogenetic tree of Eg.P29 by using the MUSCLE algorithm in MEGA11; (J) Multi-sequence of parasite species comparisons diagram. The sequence of

top six various parasite species from phylogenetic tree were selected for detailed sequence comparison and domain analysis by, ESPript3 website https:// espript. ibcp. fr/ ESPript/ ESPript/, Red dashed boxes indicate N-BAR domains, and same amino acids in different species are marked in yellow.

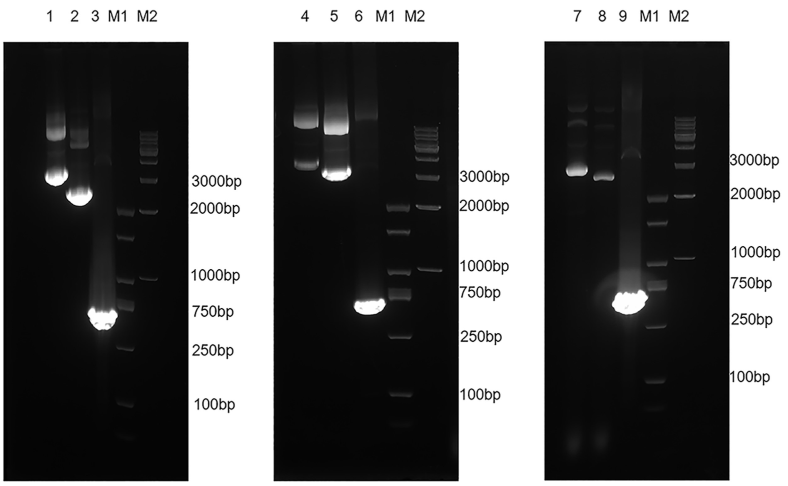

Supplementary Figure 2. Construction of *P29*/pCMV-4flag, *P29*/3HA-pCDNA3.0 and *P29*/Myc-BioID2-MCS eukaryotic expression recombinant plasmids.

Land 1 pCMV-4flag empty vector; land 2 *P29*/pCMV-4flag plasmid; land 4 3HA- pCDNA3.0 empty vector; land 5 *P29*/3HA-pCDNA3.0 plasmid; land 7 Myc-BioID2-MCS vector; land 8 *P29*/Myc-BioID2-MCS plasmid; land 3,6,9 shown *P29* amplified fragment; land M1 represents Marker of D2000 DNA standard molecular weight Ladder; land M2 represents Marker of 1kb DNA standard molecular weight Ladder.

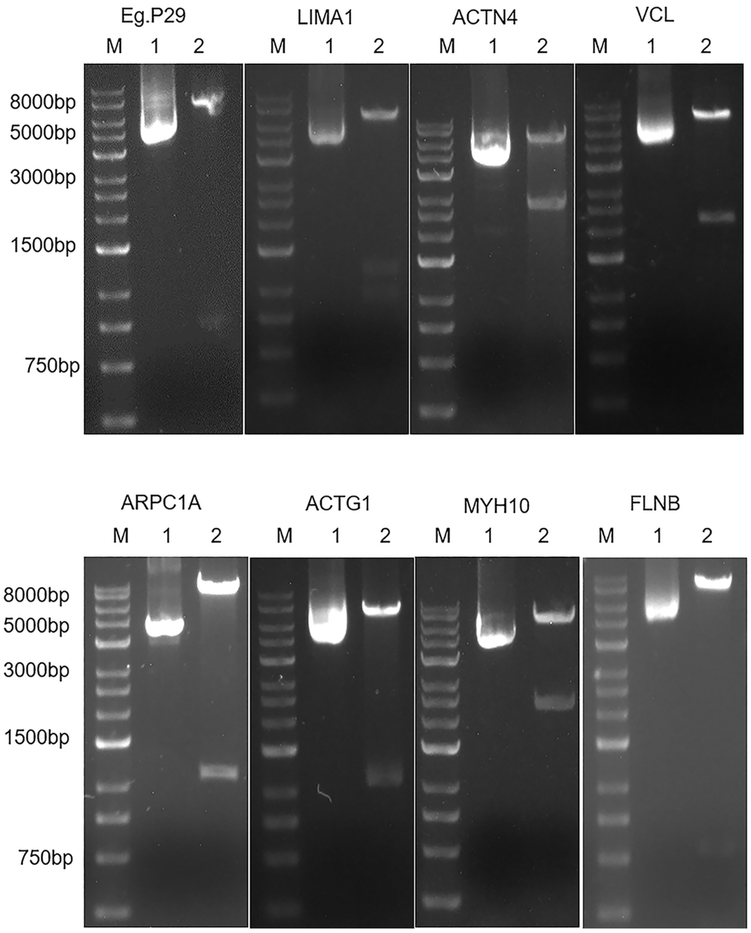

Supplementary Figure 3. Restriction Digestion Map of the bait vector and prey vector.
Lane M represents Marker of 1kb DNA standard molecular weight Ladder, Lane 1 represents Plasmid, Lane 2 represents Plasmid Digested with restriction enzyme. *Eg.P29*/pGBKT7 is bait vector ,and *ACTG1*/ pGADT7, *ACTN4*/ pGADT7, *LIMA1*/ pGADT7, *ARPC1A*/ pGADT7, *VCL*/ pGADT7, *FLNB*/ pGADT7 and *MYH10*/ pGADT7 are Prey vector.

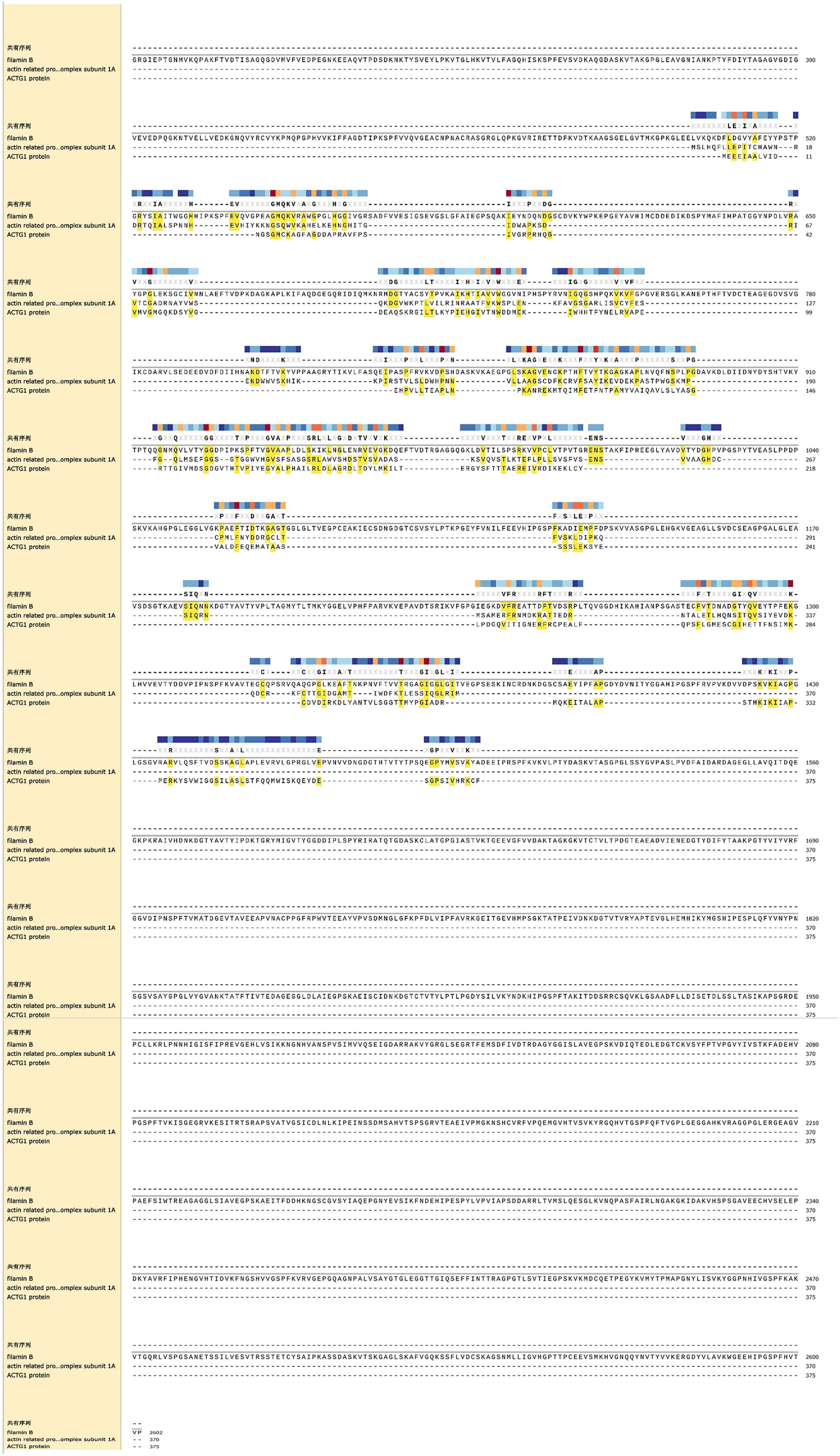

Supplementary Figure 4. Sequence alignment results of ACTG1, FLNB and ARPC1A. The amino acid sequences of ACTG1, FLNB and ARPC1A were downloaded from NCBI and imported into MEGA software for sequence comparison. The yellow markers in the figure represent amino acids that are identical in two or three of the sequences.

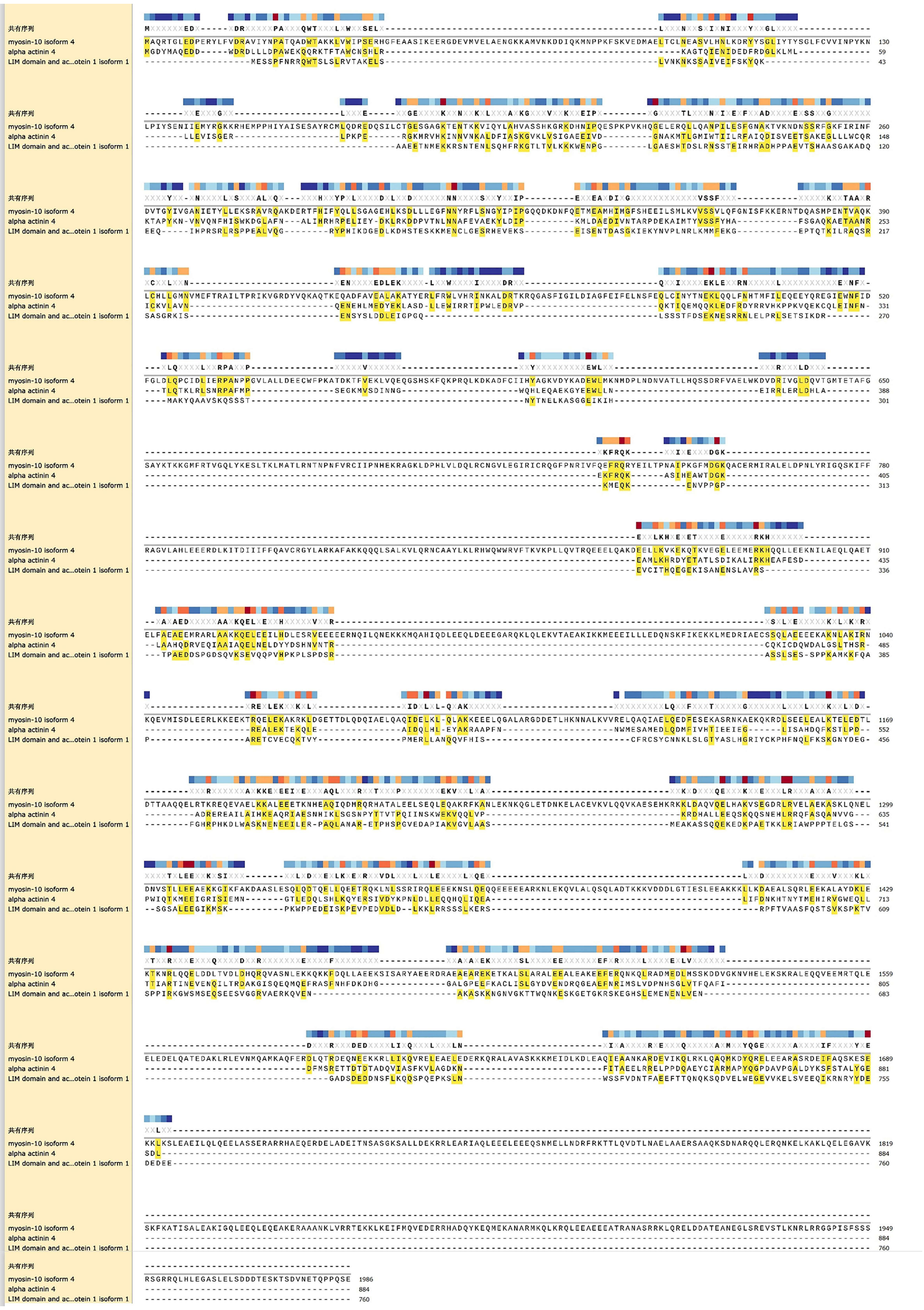

Supplementary Figure 5. Sequence alignment results of LIMA1, ACTN4 and MYH10. The amino acid sequences of LIMA1, ACTN4 and MYH10 were downloaded from NCBI and imported into MEGA software for sequence comparison. The yellow markers in the figure represent amino acids that are identical in two or three of the sequences.

3.2 Supplementary Tables

Supplemental Table S1. Major bioinformatics databases

databases	Website
ProtParam	https://www.expasy.org/resources/protparam
DeepTMHMM	https://dtu.biolib.com/DeepTMHMM
ProtScale	https://www.expasy.org/resources/protscale
SignalP6.0	https://services.healthtech.dtu.dk/services/SignalP-6.0/
NetPhos3.1	https://services.healthtech.dtu.dk/services/NetPhos-3.1/
Cell-PLoc 2.0	http://www.csbio.sjtu.edu.cn/bioinf/Cell-PLoc-2/
Prosite	https://prosite.expasy.org/
PredictProtein	https://predictprotein.org/
SMART	https://smart.embl.de/
PSIPRED	http://bioinf.cs.ucl.ac.uk/psipred/
Phyre2	http://www.sbg.bio.ic.ac.uk/phyre2/html/page.cgi?id=index
SIB Expasy	https://www.expasy.org/

Supplemental Table S2. Plasmids used in the study

Plasmid Name	Related Resistance
pEGFP-C1	Kana
pCMV-4flag	Amp
Myc-BioID2-MCS	Amp
3HA-pCDNA3.0	Amp
P29/pET-28a	Kana
P29/ pEGFP-C1	Kana
P29/ pCMV-4flag	Amp
P29/ Myc-BioID2-MCS	Amp
P29/3HA-pCDNA3.0	Amp

Supplemental Table S3. Sequences of primers involved in RT-qPCR.

Gene	Forward Primer	Reverse Primer
ACTG1	GGGAACAAAAGGCGGGGTC	ATGGAAGGAAACACGGCTCG
ACTN4	TGGAGGTCATATCAGGGGAGC	GAGACCAGCTTGACGCCTTT
LIMA1	GACTCCCAGGTTAAGAGTGAGG	TTGCAGGTGCCTGAAACTTCT
ARPC1A	ATTGCCCTCAGTCCCAATAATCA	CAAGTGACAATGCGGTCGC
VCL	CTCGTCCGGGTTGGAAAAGAG	AGTAAGGGTCTGACTGAAGCAT
FLNB	TCTTTAAAGGTGACCCGAAGGG	TCCTGAGATGGGGTGTAGGTT
MYH10	GAGTCAGGTGCTGGGAAGAC	ACTGGTTTAGGCGATTCAGGA
GAPDH	ACAACTTTGGTATCGTGGAAGG	GCCATCACGCCACAGTTTC

Supplemental Table S4. Physicochemical properties of Eg.P29.

Physicochemical properties	Predicted results
sequence	MSGFDVTKTFNRFTQRAGELVNKNEKTSYPTRTSDLIHEIDQ<br>MKAWISKIITATEEFVDINIASKVADAF<br>QKNKEKITTTDKLGTALEQVASQSEKAAPQLSKMLTEASDVH<br>QRMATARKNFNSEVNTTFIEDLKNFLNT<br>TLSEAQKAKTKLEEVRLDLDSDKTKLKNAKTAEQKAKWEA<br>EVRKDESDFDRVHQESLTIFEKTCKEFDGL<br>SVQLLDLIRAEKNYYEACAKECSMMLGE
Number of amino acids	238
Molecular weight	27096.59
Theoretical PI	5.63
Molecular formula	C1180H1906N324O386S9
Estimated half-life	30h>20h>10h
Instability index	35.16
GRAVY	-0.697

Supplemental Table S5. The LC-MS/MS result of actin-related protein of interacting with Eg.P29 protein.

Gene	Protein MW	Protein name	Num Unique	%Cov	Peptide Count
FLNB	278166.3	Filamin-B	25	15.2	22
LIMA1	85226.2	LIM domain and acti n-binding protein 1	22	24.2	12
ACTN4	104854.9	Alpha-actinin-4	7	7.1	4
ARPC1A	41569.7	Actin- related protein 2/3 co mplex subunit 1A	7	7.2	3
VCL	123800.4	Vincluin	3	5	2
ACTG1	41793.2	Actin, cytoplasmic 2	14	18	48.8
MYH10	229001	Myosin-10	28	21.6	28

Supplemental Table S6. Prediction results of subcellular localization of actin related proteins interacting with Eg.29

Gene symbol	subcellular localization
FLNB	Cytoplasm/Cytoskeleton/nucleus
LIMA1	Cytoskeleton
ACTN4	Cytoplasm/Cytoskeleton/nucleus
VCL	Cytoskeleton
ARPC1A	nucleus
ACTG1	Cytoskeleton
MYH10	Cytoplasm/Cytoskeleton/nucleus

Supplemental Table S7. Homologous molecules corresponding to seven human proteins in E. Granulosus

Homo sapiens	Domain	*Echinococcus granulosus*	Domain	Percents
LIMA1	LIM	LIM and SH3 domain protein	LIM SH3	41.18%
MYH10	Myosin N-terminal SH3-like;IQ; Myosin Motor	Myosin-11	Myosin N-terminal SH3-like; Myosin Motor	50.23%
ACTG1	Actin	Actin	Actin	96.26%
VCL		vinculin		33.80%
ARPC1A	WD40	actin related protein 2/3 complex subunit 1A:1B	WD40	50.31%
ACTN4	CH，EF_H	Alpha-actinin-2	CH; EF-hand	54.45%
FLNB	CH	filamin	CH	38.69%

Supplemental Table S8. Homology analysis between the interacting proteins

Order	Protein X	Protein Y	Idendity
1	ACTG1	ARPC1A	46.70%
2	ACTG1	FLNB	42.00%
3	LIMA1	ACTN4	45.20%
4	LIMA1	MYH10	45.10%
5	ARPC1A	FLNB	42.00%
6	ACTN4	MYH10	43.90%

4 References

Gang, R., Parimala, V., Janet S, L., Barbara, W., and Alan L, M. (2006). The BAR domain proteins: molding membranes in fission, fusion, and phagy. *Microbiol Mol Biol Rev* 70(1), 37-120. doi: 10.1128/mmbr.70.1.37-120.2006.

Maryam, R., Mehri, H., Parasoo, E., Mohammad Reza, A.K., and Saeid, B. (2024). Design and computational analysis of an effective multi-epitope vaccine candidate using subunit B of cholera toxin as a build-in adjuvant against urinary tract infections. *Bioimpacts* 14(1). doi: 10.34172/bi.2023.27513.
